# A Comparative Evaluation Framework Integrating Machine Learning and Deep Learning Models with ADME-Based Pharmacokinetic Assessment for HIV-Related Compounds

**DOI:** 10.3390/ph19081267

**Published:** 2026-08-11

**Authors:** Bihter Das, Harun Uslu, Ayca Bostancioglu, Bunyamin Goktas, Yunus Santur, Seval Yilmaz, İbrahim Türkoğlu

**Affiliations:** 1 Department of Software Engineering, Faculty of Technology, Firat University, 23119 Elazig, Türkiye; aycabostancioglu@gmail.com (A.B.); iturkoglu@firat.edu.tr (İ.T.); 2 Faculty of Pharmacy, Firat University, 23119 Elazig, Türkiye; huslu@firat.edu.tr (H.U.); bgoktas@firat.edu.tr (B.G.); 3 Graduate School, Anadolu University, 26470 Eskisehir, Türkiye; 4 Department of Artificial Intelligence and Data Engineering, Faculty of Engineering, Firat University, 23119 Elazig, Türkiye; ysantur@firat.edu.tr; 5 Department of Biochemistry, Faculty of Veterinary Medicine, Firat University, 23119 Elazig, Türkiye; sevalyilmaz@firat.edu.tr

**Keywords:** artificial intelligence, HIV inhibitors, molecular property prediction, molecular docking, drug discovery, ADME prediction

## Abstract

**Background/Objectives:** Predicting the bioactivity of HIV-related compounds is essential for early-stage drug discovery. However, most existing machine learning (ML) studies emphasize predictive performance while overlooking the predicted pharmacokinetic and drug-likeness properties of prioritized compounds. This study presents a comparative framework integrating classical ML, deep learning, graph-based models, and complementary ADME-based pharmacokinetic assessment. **Methods:** Twelve predictive models were evaluated using stratified five-fold cross-validation on the MoleculeNet HIV dataset under a unified experimental protocol. Model performance was assessed using multiple classification metrics together with statistical analysis. The highest-ranked compounds from the independent test set were further characterized using predicted ADME and drug-likeness properties. A representative compound (GDL1), prioritized by the GDL model, was subsequently evaluated by molecular docking against HIV-1 protease, HIV-1 integrase, and HIV-1 reverse transcriptase. **Results:** The graph-based GDL model achieved the highest ROC–AUC (0.956±0.015), followed by GRU (0.930±0.017) and RF (0.927±0.023). Statistical analysis indicated overall differences among model performances (Friedman test, p<0.001). However, Holm-corrected pairwise comparisons did not demonstrate statistically significant differences between the highest-performing models. Comparative ADME analysis showed that high predictive performance did not necessarily correspond to favorable predicted pharmacokinetic properties. Molecular docking suggested potential predicted binding interactions of the prioritized GDL1 compound with all three HIV-1 targets, with the most favorable predicted binding affinity observed for HIV-1 reverse transcriptase. **Conclusions:** The proposed framework enables a comprehensive comparison of diverse molecular learning approaches by integrating predictive performance with complementary predicted ADME, drug-likeness, and molecular docking analyses.

## 1. Introduction

Traditional drug discovery and development processes, despite advanced technological developments, remain quite costly, time-consuming, and high-risk. Bringing a new drug through clinical trials to market takes many years, and high failure rates in this process lead to significant economic losses [1]. To overcome these challenges, computational chemistry, bioinformatics, and Computer-Aided Drug Design (CADD) approaches have become critical tools for accelerating drug discovery processes and minimizing costs [2]. Human Immunodeficiency Virus (HIV)/Acquired Immunodeficiency Syndrome (AIDS), affecting approximately 40 million people worldwide and for which there is no definitive cure or vaccine yet, is one of the most important battlegrounds of modern medicine. Current antiretroviral therapies cannot eliminate the virus; they only suppress viral replication, extending patients’ lifespan. This necessitates lifelong medication for patients, negatively impacting both their individual quality of life and the economic burden on healthcare systems. Therefore, the discovery of more effective and pharmacologically appropriate anti-HIV molecules is of great importance [3,4].

In recent years, artificial intelligence, and especially deep learning methods, have made significant progress in modeling molecular properties and predicting biological activity [5]. In this context, graph-based geometric deep learning (GDL) approaches model molecular structures as atom–bond graphs and learn expressive molecular representations through graph message-passing operations, offering the potential to capture molecular topology more effectively than traditional feature-engineering-based machine learning methods [6,7,8]. Beyond molecular modeling, hybrid deep learning frameworks that combine complementary architectures—such as vision transformers and graph convolutional networks—have also demonstrated strong performance in biomedical diagnosis, further underscoring the broader utility of graph-based learning in biomedical applications [9]. However, the literature shows that different model families are often evaluated under different data representations, different evaluation metrics, and inconsistent experimental protocols [10,11]. This makes it difficult to make a sound comparison of the actual performance and generalizability of the models. Within the same bioactivity problem, sparse feature representations obtained with Morgan (circular) fingerprints, learned embedding structures based on SMILES character strings, and representation spaces learned from atom-bond graphs have different inductive bias properties [12,13]. This diversity leads to models learning the chemical space in different ways; however, the lack of evaluation of these models within a common experimental framework limits the interpretability of the results obtained. Furthermore, class imbalance, the Precision–Recall balance is sensitive to threshold selection in sigmoid outputs, and evaluations based solely on limited metrics such as accuracy or ROC–AUC lead to incomplete analysis of model performance [14]. However, a large portion of current studies focus only on prediction performance; the evaluation of model outputs in terms of pharmacokinetic fitness (ADME) and drug-likeness is often neglected [15,16]. Yet, in early-stage drug discovery processes, not only high-performance predictive models but also approaches capable of prioritizing compounds with favorable predicted pharmacokinetic and drug-likeness properties are critical. Criteria such as the Lipinski rules, QED, and TPSA provide useful preliminary indicators for evaluating the drug-likeness and predicted pharmacokinetic characteristics of candidate compounds during the early stages of drug discovery [17,18,19].

This study aims to address the aforementioned gaps by presenting a comprehensive model comparison for predicting the molecular bioactivity of HIV. In this study, classical machine learning methods (SVM, RF, MLP, and Mol2Vec+SVM models), sequential deep learning approaches (RNN, GRU, BRNN, CNN), and graph-based GDL models (GCN, GAT, MPNN, and GDL model) were evaluated under a single standard experimental protocol. All models were tested with five-fold cross-validation on the same data splits, and their performance was analyzed across multiple metrics. Additionally, using the SwissADME platform, we assessed the drug-likeness, physicochemical properties, and predicted pharmacokinetic profiles of the highest-ranked compounds from the independent test set identified by each model. The overall workflow of the proposed methodology, from molecular data preparation to comparative model evaluation and ADME-based characterization, is summarized in Section 3. This study explores the relationship between predictive performance and the predicted pharmacokinetic and drug-likeness properties of the prioritized compounds. The presented ADME analysis provides a descriptive characterization of selected compounds from the independent test set and should not be interpreted as evidence that any model systematically prioritizes pharmacologically superior molecules.

### 1.1. Problem Statement

The high cost of existing drugs used in the treatment of HIV/AIDS, the need for long-term use, and the potential for the virus to develop resistance necessitate the development of more effective and sustainable inhibitors [3,4]. Experimental bottlenecks and high costs encountered in traditional drug discovery processes have led researchers to high-throughput virtual screening and machine learning-based approaches [20,21,22,23]. However, a significant portion of current methods are limited to table-level descriptors or single molecular representations, leading to insufficient modeling of structural dependencies at the graph and sequence levels [10].

However, a molecule demonstrating biological activity alone is not sufficient for it to be considered a drug candidate. Candidate molecules must also be pharmacokinetically suitable and meet drug-likeness criteria. In this context, criteria such as Lipinski’s Rule of Five, QED, and TPSA provide key indicators for evaluating the oral bioavailability and developmental potential of molecules [15,17]. However, many studies in the literature focus on prediction performance and do not systematically integrate such pharmacokinetic criteria into the model evaluation process [16,23].

The central problem addressed in this study is the lack of a holistic analysis framework that enables transparent and fair comparison among different molecular representations (fingerprint, sequence, and graph-based approaches) and learning architectures, while also prioritizing high-confidence compounds based on their predicted pharmacokinetic and drug-likeness characteristics.

The vast majority of studies in the literature on HIV bioactivity prediction present with a single model architecture or a limited number of algorithms; data preprocessing protocols, cross-validation coefficients, and hyperparameter selection criteria are reported inconsistently from study to study [10,11]. This methodological heterogeneity makes it difficult to directly compare results even when using a common reference data frame and limits the generalizability of the findings.

Therefore, the fundamental problem is not simply improving the predictive performance of a single model. Rather, the real deficiency is the lack of a reproducible and transparent experimental framework in which multiple model families can be compared under the same experimental protocol, inter-fold variability and model uncertainty can be assessed, and model outputs can be evaluated not only using predictive performance metrics but also through predicted ADME and drug-likeness assessments.

### 1.2. Main Contributions

This study aims to systematically and statistically evaluate the relative performance of different molecular representations and learning architectures on predicting the molecular bioactivity of HIV under the same experimental protocol, instead of the “one best model” approach commonly found in the literature [5,11,22,24,25]. Using the HIV dataset provided under the MoleculeNet framework, standardized preprocessing at the SMILES level was first applied, and basic physicochemical descriptors (MW, LogP, HBD, HBA, TPSA, number of rotatable bonds, QED) were calculated via RDKit. Furthermore, the predicted pharmacokinetic and drug-likeness characteristics of the prioritized compounds were systematically assessed using Lipinski rules and related descriptors, thereby enabling model evaluation based not only on predictive performance but also on complementary molecular property assessments.

The methodological framework encompasses three fundamental representation paradigms: (i) classical machine learning models (RF, SVM, MLP) based on Morgan (circular) fingerprint, together with a Mol2Vec+SVM model using Mol2Vec molecular embeddings; (ii) sequence-based deep learning architectures (RNN, BRNN, CNN, GRU) with embedding representations learned from SMILES sequences; and (iii) graph neural networks (GCN, GAT, MPNN, and GDL) operating on atom–bond graphs. All models were trained using five-fold cross-validation under identical data splits; multiple performance metrics such as Precision, Recall, $F_{1}$, and ROC–AUC were reported along with mean ± standard deviation [14]. Furthermore, the statistical significance of inter-model differences was analyzed using the Friedman test and multiple comparison corrections. This study offers the following scientific and technical contributions:

- By comparing different types of molecular representation (fingerprint, SMILES, graph) and model families under a single standard experimental protocol, a systematic and reproducible evaluation framework for model selection and representation preference is presented.
- Model performance is evaluated not only through predictive performance metrics but also through predicted ADME and drug-likeness assessments based on Lipinski rules and physicochemical descriptors, enabling the prioritization and comparative characterization of high-confidence compounds from the independent test set.
- When the predicted QED score, ADME profile, and molecular docking results were considered together, the selected GDL1 compound exhibited favorable predicted drug-likeness properties and favorable predicted docking scores against HIV-1 integrase, HIV-1 protease, and HIV-1 reverse transcriptase.
- The significance of inter-model differences is demonstrated not only observationally but also statistically through five-fold cross-validation, mean ± standard deviation reporting, and advanced statistical tests (Friedman, post hoc analyses, and effect size measurements).
- The evaluation of twelve different model families under the same data and protocol allows for a direct comparison of the strengths and weaknesses of different representation approaches.

The rest of the article is structured as follows: Section 1.3 summarizes relevant studies in the field of molecular bioactivity prediction of HIV. Section 2 presents experimental results and model comparisons, discussing findings with performance curves, statistical analyses, ADME, and docking analysis. Section 3 includes detailed descriptions of data collection and preprocessing, physicochemical descriptors, Lipinski rules, evaluation metrics, and model architectures used. Finally, Section 4 presents a general evaluation of the study and directions for future research.

### 1.3. Literature Review

Computational methods have played an increasingly decisive role in the discovery of drugs against HIV over the past decade [5]. Considering the high cost and long duration of traditional trial-and-error-based drug development processes, the integration of machine learning and deep learning techniques into bioactivity prediction, virtual screening, and de novo molecular design has gained significant momentum [2]. In particular, the application of SMILES-based molecular representations, fingerprint descriptors, and modern approaches such as GNN to HIV-targeted studies has yielded promising results in both the classification of existing inhibitors and the generation of new candidate compounds [7,25]. In this section, studies conducted in the field of HIV molecular bioactivity prediction are reviewed, and general trends and research gaps in the literature are discussed. A summary of representative studies on HIV molecular bioactivity prediction, including their datasets, methods, and key findings, is provided in Table 1.

Li et al. (2018) developed 15 classification models, including k-NN, decision trees, RF, SVM, and DNN, on a dataset of 4855 HIV-1 protease inhibitors compiled from the ChEMBL database [34]. The consensus model based on MACCS fingerprint descriptors achieved an accuracy of 91.96% on the training set and 83.15% on the test set. The strengths of this study include the use of an extensive external validation set and the comparison of multiple descriptor types; however, the lack of a dedicated mechanism to address class imbalance is considered a limitation. Machado et al. (2022) aimed to identify natural compounds targeting the HIV-1 integrase enzyme using a machine learning-based ligand-based virtual screening approach [21]. Comprehensive hyperparameter optimization was performed to address the class imbalance problem, and the best-performing model was used to screen active compounds from the Natural Product Atlas. The applicability domain analysis provided a strong methodological contribution by distinguishing between “predictable” and “unknown” regions of the chemical space; however, the absence of in vitro validation limits the confirmation of the predicted biological activity.

Phan et al. (2024) compared 16 different fingerprint and molecular descriptor types using the Wilcoxon signed-rank test for the classification of HIV-1 integrase inhibitors and subsequently evaluated 10 machine learning models using cross-validation [35]. The best model achieved an F1 score of 0.889 and an average precision of 0.921 on the external validation set. Although the study provides a comprehensive pipeline integrating applicability domain analysis and DrugBank repurposing data, the exclusion of deep learning architectures limits a full comparison with graph-based methods. Das et al. (2022) developed a GDL-based MPNN model to predict drug–target interactions for HIV by transforming SMILES-based drug data into molecular and subsequently graph representations, achieving an accuracy of 93.3% [36]. A notable strength of the study is the provision of an interpretable interface that visually demonstrates how molecules bind to targets. However, the relatively small dataset and the lack of generalization across different HIV proteins represent important limitations. Kutsal et al. (2024) employed a customized LSTM-based variational autoencoder architecture to generate novel HIV drug candidates from SMILES-encoded compounds, achieving 91% training accuracy on a dataset of 1377 compounds [37]. The generated candidate molecules were validated in terms of Lipinski’s Rule of Five compliance, and their interaction potentials were evaluated using a previously developed artificial intelligence model. However, the relatively small dataset and the lack of in vitro or in vivo validation limit the reliability of the generated candidates.

Uslu et al. (2025) proposed a three-stage integrated artificial intelligence framework combining autoencoder-LSTM-based de novo molecule generation, geometric deep learning-based interaction prediction, and molecular docking validation to accelerate the identification of novel therapeutic compounds against HIV [16]. The developed system provides an end-to-end workflow, from generating pharmacokinetically suitable compounds to predicting their binding affinities with target proteins. However, the absence of large-scale prospective evaluations limits the assessment of its clinical applicability. The approach referred to as MPNN-CWExplainer was built upon an MPNN framework addressing the class imbalance problem in the MoleculeNet HIV dataset through a class-weighted loss function, followed by atom- and bond-level interpretability using GNNExplainer [25]. The model outperformed baseline methods with an AUC-ROC of 87.63% and an AUC-PRC of 86.02%. However, a key limitation is that GNNExplainer, as a post hoc interpretability method, captures correlations rather than true causal relationships, which requires careful interpretation in drug design decisions. In another study focusing on HIV-1/HBV co-infection, an ensemble model combining DMPNN and GBDT was developed to perform multi-target bioactivity prediction across 12 critical HIV-1 and HBV targets [38]. The model successfully identified potential multi-target profiles for 22 approved HIV-1 drugs. While its ability to perform simultaneous multi-target prediction is a key strength, further systematic validation is required to assess the consistency between model predictions and experimentally verified data for each target.

Existing studies reveal significant progress in the field of HIV bioactivity prediction, but three key shortcomings stand out. Firstly, the vast majority of studies are conducted using a single model architecture or a limited number of algorithms, and systematic comparisons of different representation approaches are lacking. Secondly, inconsistencies exist in data splitting, evaluation metrics, and experimental protocols, making fair comparisons between models difficult. Thirdly, and most critically, the integration of predicted ADME properties and drug-likeness assessments into the comparative evaluation of model outputs remains largely unexplored. Given these shortcomings, there is a need for a holistic and standardized evaluation framework that enables fair comparison across multiple model families while integrating predictive performance with complementary assessments of predicted ADME and drug-likeness properties. This study addresses this gap by comparing different molecular representations and learning approaches under a unified experimental protocol and by performing comparative characterization of the highest-ranked compounds from the independent test set using predicted ADME and drug-likeness assessments.

## 2. Experimental Results and Discussion

This section presents a comprehensive evaluation of the experimental framework developed for predicting HIV bioactivity. The study compares a broad family of models, including classical machine learning models (RF, SVM, MLP, Mol2Vec+SVM), sequence-based deep learning models (BRNN, CNN, GRU, RNN), and graph-based models (GCN, GAT, the graph-based GDL model, and MPNN), under the same data partitioning strategy (72% training, 10% validation, and 18% independent testing) and a unified experimental protocol. Model development was performed using the training subset with five-fold cross-validation, while the independent test set was reserved exclusively for the final evaluation. The independent hold-out test set was not used for model selection, hyperparameter tuning, fold selection, or any intermediate performance comparison. It was accessed only after completion of model development for the final independent evaluation and compound prioritization. An early stopping mechanism was implemented for all deep learning models to prevent overfitting and improve generalization. Training was terminated when the validation loss did not improve for a predefined patience value. This strategy ensured a consistent and fair evaluation framework across all models.

This study extends a conventional comparison of predictive performance by adopting a multi-stage evaluation framework. In the first stage, model performance was quantitatively evaluated using multiple classification metrics and graphical analyses. In the second stage, the statistical significance of the observed performance differences between the models was assessed using complementary statistical tests. In the final stage, the two highest-ranked compounds from the independent test set identified by each model, based on their predicted probabilities, were comparatively characterized using predicted ADME and drug-likeness properties. This complementary analysis was performed to investigate whether compounds prioritized by different predictive models exhibited distinct predicted molecular characteristics. Consequently, the study examines the relationship between predictive performance and the predicted physicochemical and drug-likeness profiles of the prioritized compounds rather than making claims regarding experimental efficacy, pharmacological suitability, or clinical applicability.

### 2.1. Performance Evaluation of the Models

This section presents a comparative performance analysis of classical machine learning, sequence-based deep learning, and graph-based deep learning models evaluated within the scope of the HIV bioactivity prediction problem. All models were evaluated using the mean and standard deviation (mean ± std) values of five-fold cross-validation results, and the obtained results are given in Table 2. These cross-validation performance metrics were used exclusively for comparative model evaluation and should not be confused with the independent hold-out test predictions used later for compound prioritization Table 3.

Looking at the performance comparison in Table 2, the graph-based GDL model exhibits the highest discrimination performance among all models with a ROC–AUC value of 0.9560 ± 0.0154. However, while the GDL model offers high accuracy in positive class prediction with a precision value of 0.9966 ± 0.0077, its relatively lower recall value (0.7480 ± 0.0513) indicates that the model has a more conservative decision-making mechanism. Among deep learning models, the GRU model stands out as one of the most balanced and high-performing models with accuracy values of 0.892 ± 0.019 and ROC–AUC values of 0.930 ± 0.017. GRU’s balanced performance in both precision (0.909 ± 0.056) and recall (0.821 ± 0.028) indicates that the model is successful in both capturing true positives and minimizing false positives. When machine learning models are examined, RF and SVM models are seen to offer strong and stable performances. The RF model stands out as the best classical model with a ROC–AUC value of 0.9274 ± 0.0227, while the SVM model shows similarly high discrimination performance with a ROC–AUC value of 0.9153 ± 0.0178. However, the Mol2Vec+SVM pipeline achieved competitive performance by leveraging learned molecular embedding representations, highlighting the benefit of data-driven molecular feature extraction over handcrafted descriptors. When graph-based models (GCN, GAT, MPNN) are evaluated, it is observed that these models exhibit lower performance compared to classical machine learning methods. Specifically, the MPNN model’s ROC–AUC value of 0.729 ± 0.042 indicates that graph representation-based learning provides a limited advantage in this dataset. This difference may be associated with differences in model architecture, selected hyperparameter settings, or characteristics of the dataset. When sequence-based deep learning models (CNN, RNN, and BRNN) are examined, it is seen that these models generally exhibit low performance. The ROC–AUC values of the CNN and BRNN models remaining at 0.5120 ± 0.0168 and 0.4909 ± 0.0256, respectively, show that learning from the raw SMILES sequences does not adequately capture molecular structural dependencies. In the RNN model, a high variance (±0.220 accuracy) is noteworthy, indicating that the model has unstable learning dynamics.

**Figure 1:**
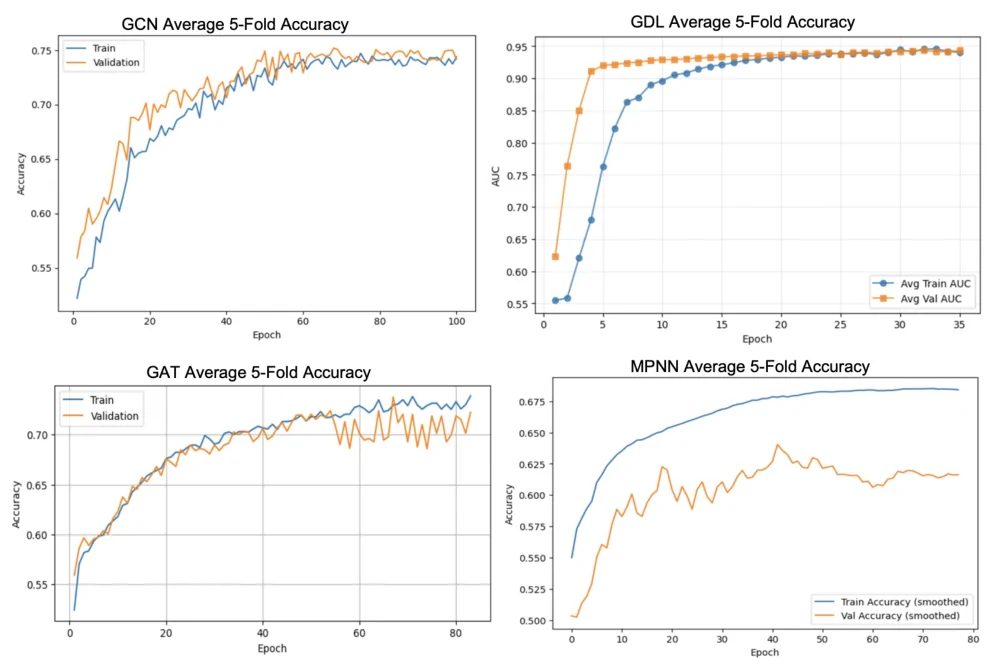

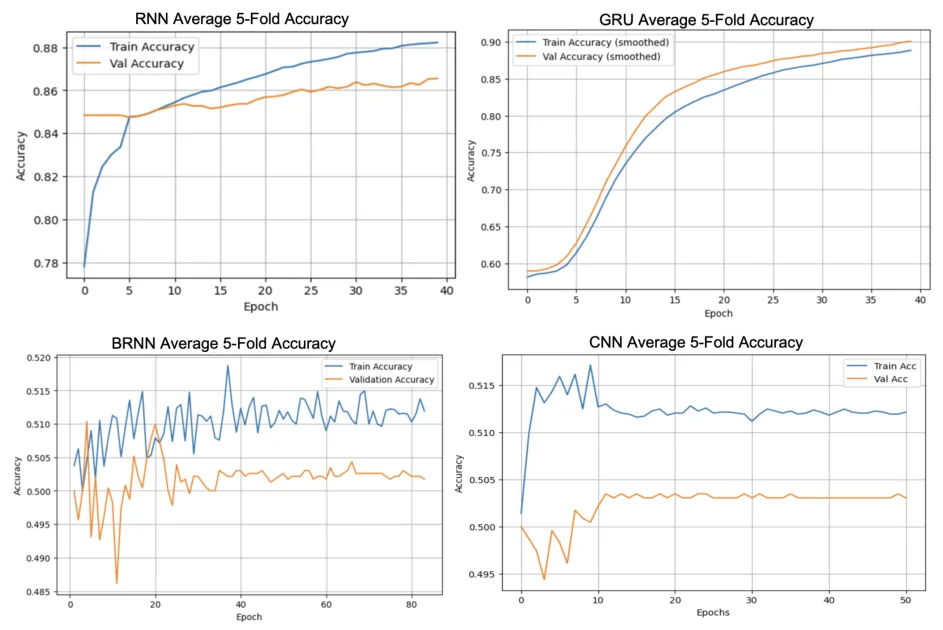
Training and validation accuracy curves of the deep learning and graph-based models from a representative fold of the five-fold cross-validation.

**Figure 2:**
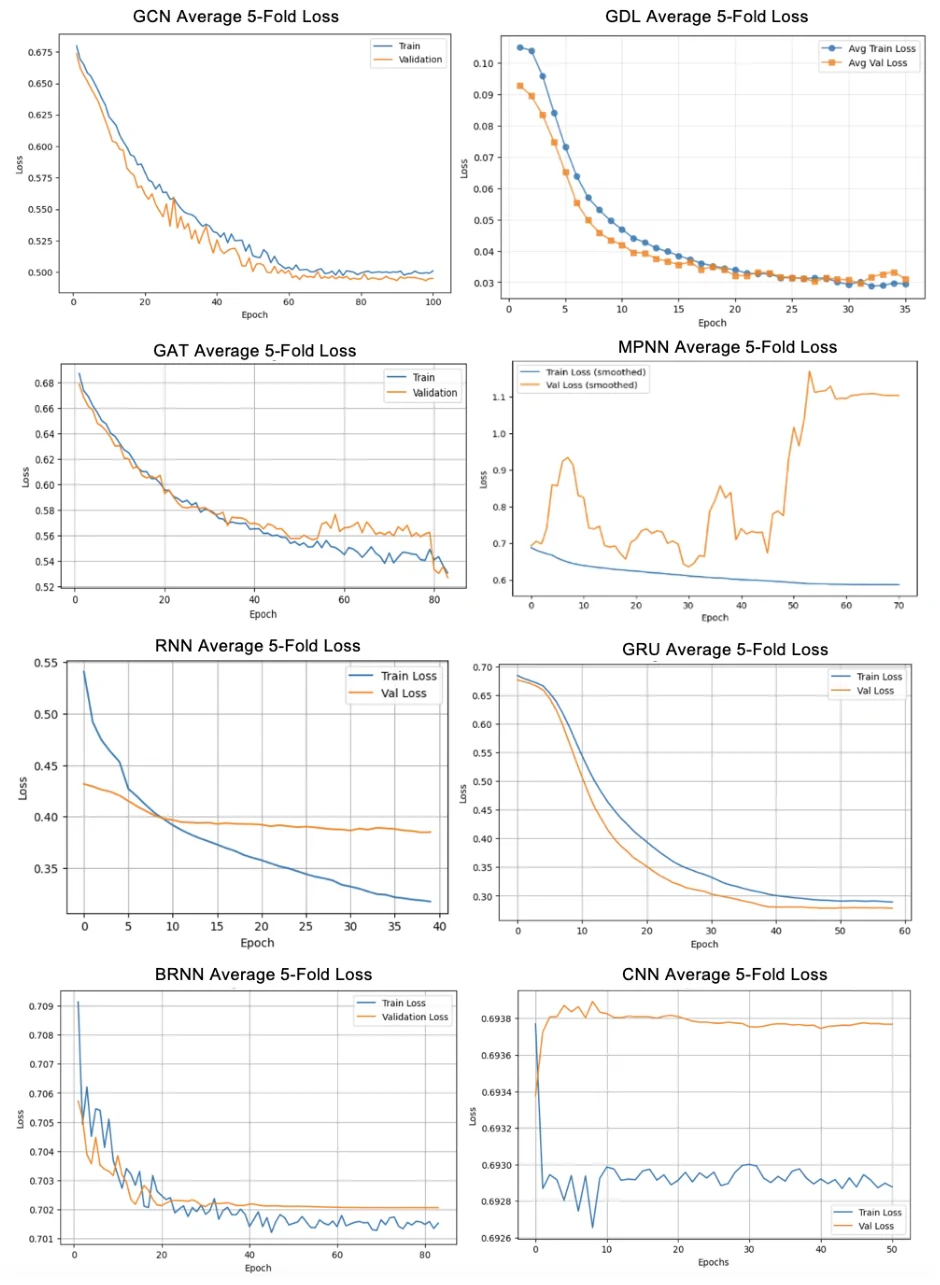
Training and validation loss curves of the deep learning and graph-based models from a representative fold of the five-fold cross-validation.

Examining the accuracy and loss curves given in Figure 1 and Figure 2, it is observed that high-performance models exhibit more stable and faster convergence behavior. In contrast, it has been observed that the learning process is more fluctuating in low-performance models and their generalization ability remains limited.

**Figure 3:**
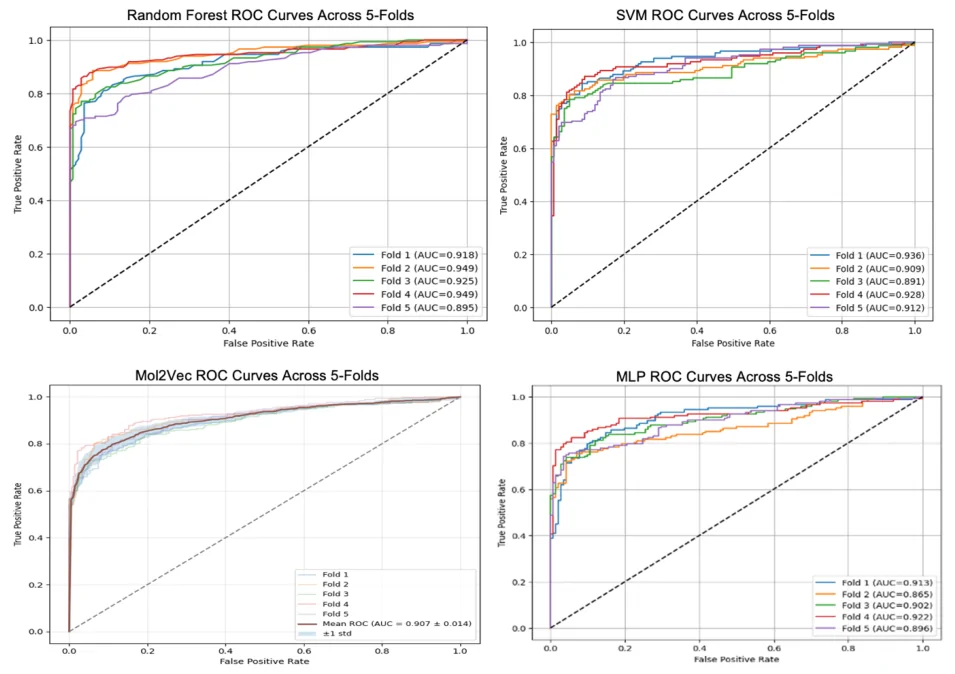
ROC curves of machine learning models for 5-fold cross validation.

The ROC curves presented in Figure 3 clearly show that the GRU, RF, and GDL models have higher discrimination capacity compared to other models. Overall, the results indicate that different model families contribute to the HIV bioactivity prediction problem at different levels. Geometric deep learning and advanced sequential models (GRU) offer the highest performance, while classical machine learning methods continue to produce stable and competitive results. In contrast, the limited performance of models based on the raw SMILES sequence highlights the critical importance of appropriate feature extraction in molecular representation.

To ensure the transparency and reproducibility of the compound prioritization procedure, Table 3 summarizes the two highest-ranked compounds selected by each predictive model for the subsequent comparative ADME analysis. For each predictive architecture, model selection was performed exclusively within the development data using five-fold cross-validation. The cross-validation fold achieving the highest validation ROC–AUC was selected as the reference model for that architecture. After model selection had been completed, the corresponding trained model was applied once to the independent hold-out test set, which had remained completely isolated throughout model development. The compounds in the hold-out test set were then ranked according to their predicted probabilities. In addition to the prediction outcomes, the corresponding cross-validation fold and Lipinski rule assessment are reported. The predicted probabilities presented in this table reflect only the confidence scores of the selected compounds and should not be interpreted as overall model performance, which is evaluated separately using the complete cross-validation results. The performance statistics reported in Table 2 summarize the five-fold cross-validation results obtained during model development, whereas the compound ranking presented in Table 3 is based solely on predictions generated for the independent hold-out test set after model selection had been completed.

To assess the consistency of compound prioritization across predictive models, an overlap analysis was performed using the canonical SMILES representations of the highest-ranked compounds selected by each model. As shown in Table 4, only two compounds were prioritized by more than one predictive model. The compound with the canonical SMILES. O=[N+]([O-])c1ccccc1S(=O)(=O)c1cc(Cl)cc2c1NCCC2 was independently selected by the SVM, MLP, and GCN models, whereas CCNc1ccccc1S(=O)(=O)c1ccccc1[N+](=O)[O-] was jointly selected by the SVM and MLP models. All remaining prioritized compounds were unique to their corresponding predictive models, indicating that different machine learning and deep learning algorithms generally prioritized different compounds from the independent hold-out test set despite being trained and evaluated on the same benchmark dataset.

To further investigate whether the highest-ranked compounds represented predictions closely related to the training data or extended beyond the nearest structural analogues, an additional fingerprint similarity analysis was performed. For each selected compound, the maximum Morgan fingerprint (radius = 2, 2048 bits) Tanimoto similarity to the compounds in the corresponding training partition was calculated, and the experimental activity label of the nearest training analogue was recorded. The results are summarized in Table 5.

As shown in Table 5, the selected compounds exhibited varying degrees of structural similarity to the corresponding training compounds. Several models prioritized compounds with high structural similarity to known training chemotypes, whereas others also identified compounds with moderate or relatively lower similarity values. In particular, the graph-based GDL model selected compounds with maximum Tanimoto similarity values of 0.556 and 0.778, indicating that its highest-ranked predictions were not restricted to the closest structural analogues present in the training data. Similarly, MLP, SVM, BRNN, and RNN also selected at least one compound with moderate or relatively low structural similarity. Overall, these findings suggest that high prediction confidence was not uniformly associated with the closest structural analogues in the training set and provide additional insight into the structural diversity of the compounds prioritized by the evaluated models.

### 2.2. Sensitivity Analysis of Inactive Subset Selection

In this study, to further evaluate whether the predictive performance depended on the specific inactive compounds contained in the archived HIV subset, an additional sensitivity analysis was performed using chemically matched alternative inactive training subsets. Thirty independent inactive subsets were generated from the MoleculeNet HIV inactive pool using Morgan fingerprints (radius = 2, 2048-bit) and Tanimoto similarity matching while preserving one-to-one correspondence with the archived HIV inactive compounds and preferentially selecting molecules sharing the same Bemis–Murcko scaffold whenever available. During this analysis, the active training compounds, held-out test set, molecular representation, Random Forest hyperparameter configuration, decision threshold, and random seed (Seed = 42) were kept identical to the original experimental protocol. Therefore, only the composition of the inactive training subset varied across repeated experiments.

The ROC–AUC values reported in this sensitivity analysis are not directly comparable with the five-fold cross-validation results presented in Table 2. Table 2 summarizes the mean ROC–AUC values obtained during five-fold cross-validation on the development set, whereas the values reported in Table 6 were obtained from an independent sensitivity-analysis protocol in which chemically matched inactive subsets were generated while keeping the held-out evaluation framework unchanged. Consequently, these numerical values arise from different evaluation procedures and should not be interpreted as alternative estimates of the cross-validation performance.

Table 6 summarizes the predictive performance obtained from 30 chemically matched inactive subsets, all using an identical experimental protocol. Mean values, standard deviations, and bootstrap confidence intervals indicate that the Random Forest classifier produced consistent predictive performance across repeated chemically constrained sampling experiments.

**Figure 4:**
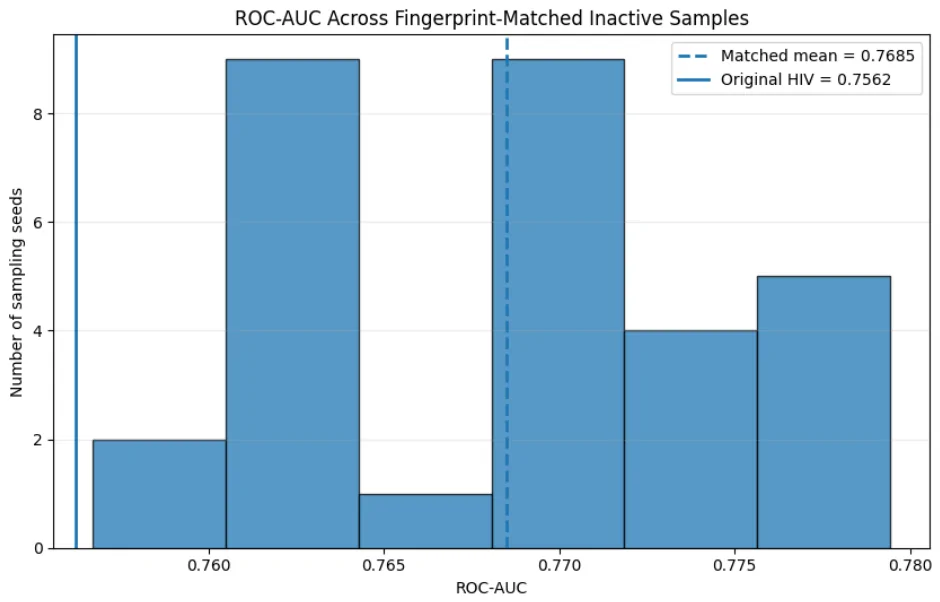
Distribution of ROC–AUC values obtained from thirty chemically matched inactive subsets.

As seen in Figure 4, the ROC–AUC values obtained across thirty independent repetitions were concentrated within a relatively narrow interval, demonstrating limited variability under chemically matched inactive sampling. The HIV inactive subset produced a comparable level of predictive performance under the same experimental protocol.

Table 7 summarizes the chemical similarity between the HIV inactive subset and the fingerprint-matched alternative subsets generated during the sensitivity analysis. The average Tanimoto similarity of 0.4856 indicates that the matched compounds retained substantial structural similarity to their corresponding molecules. Furthermore, approximately 63% of the matched compounds shared the same Bemis–Murcko scaffold, confirming that the alternative inactive subsets were chemically comparable rather than being generated through arbitrary random sampling. Consequently, the observed performance variation reflects the influence of chemically comparable inactive-compound compositions rather than differences arising from unrelated or randomly selected molecules. In this study, the sensitivity analysis demonstrates that the Random Forest classifier maintains consistent predictive behavior when the archived inactive compounds are replaced by chemically comparable alternatives. Although modest performance differences were observed across alternative inactive subsets, the overall predictive characteristics of the model remained stable under an otherwise identical experimental protocol. These findings indicate that the principal conclusions of this study are not attributable to a single arbitrary inactive subset.

### 2.3. Statistical Comparison of Models

**Figure 5:**
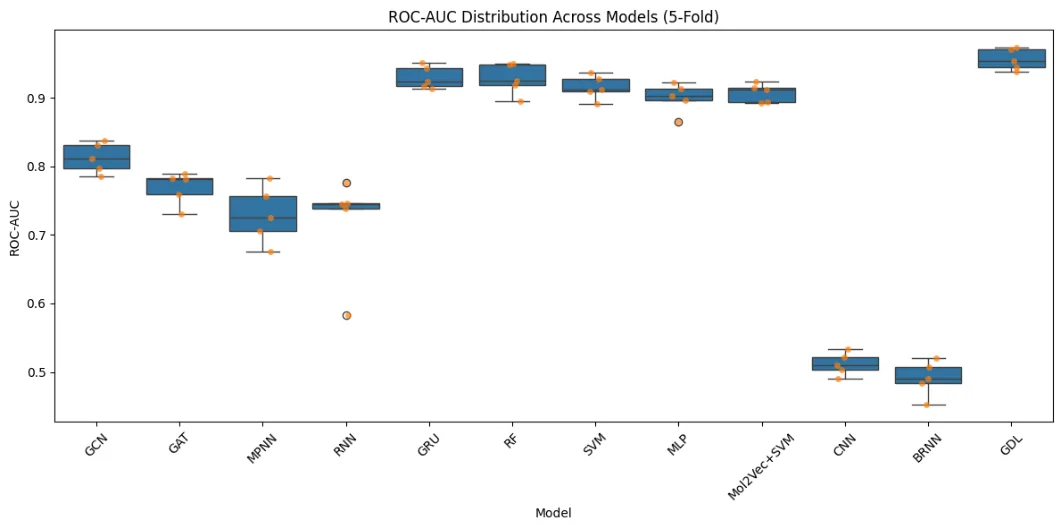
Boxplot showing 5-fold cross-validation ROC–AUC distributions for all models.

This section analyzes whether the observed performance differences between the models are statistically significant. For this purpose, a general comparison was first made on the ROC–AUC values of all models, and then pairwise comparisons were performed using the model showing the highest performance as a reference. The Friedman test was applied to test for the existence of a general performance difference between the models. The results obtained (Friedman statistic = 51.615, *p* < 0.001) clearly show that there are statistically significant differences between the models in terms of ROC–AUC. This finding reveals that model performances are not solely due to random variations but also contain significant differences. The fold-based performance distributions of the models are presented in Figure 5, and these distributions visually support the performance divergence between different model groups.

Figure 5 visualizes the performance variance and stability of the models, showing that the GDL, GRU, RF, and SVM models exhibit stable performance with both high and low variance. After the omnibus test result was found to be significant, a post hoc analysis was performed to determine which models exhibited these differences. In this stage, the GDL model, which exhibited the highest performance in terms of average ROC–AUC value, was selected as the reference model, and paired comparisons were made with all other models. This approach aims to statistically evaluate whether the observed performance differences between the best-performing model and the remaining models are significant. In pairwise comparisons, a paired *t*-test was used, and Holm correction was applied to control for the multiple comparison problem. The obtained statistical results are presented in detail in Table 8.

**Figure 6:**
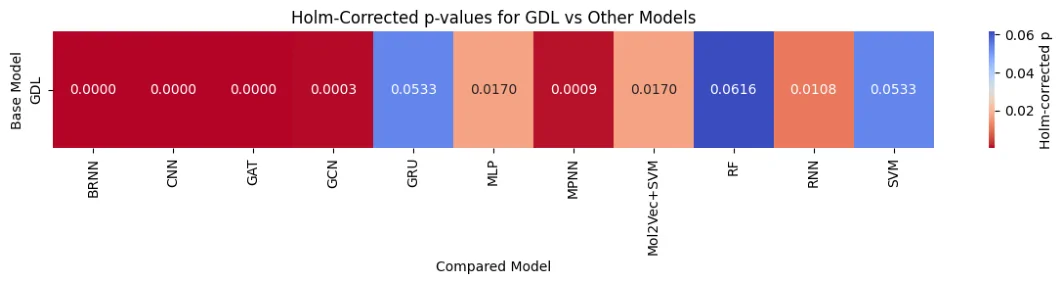
Heat map showing Holm-corrected p-values between the GDL model and other models.

When examining Table 8, it is seen that the GDL model exhibits statistically significant higher performance with *p* < 0.05 after Holm correction against the BRNN, CNN, RNN, MPNN, GAT, GCN, MLP, and Mol2Vec+SVM models. This finding is also visually supported by the Holm-corrected p-value heatmap presented in Figure 6. The intense color regions corresponding to low p-values in the heatmap clearly reveal the strong statistical differences between these models.

**Figure 7:**
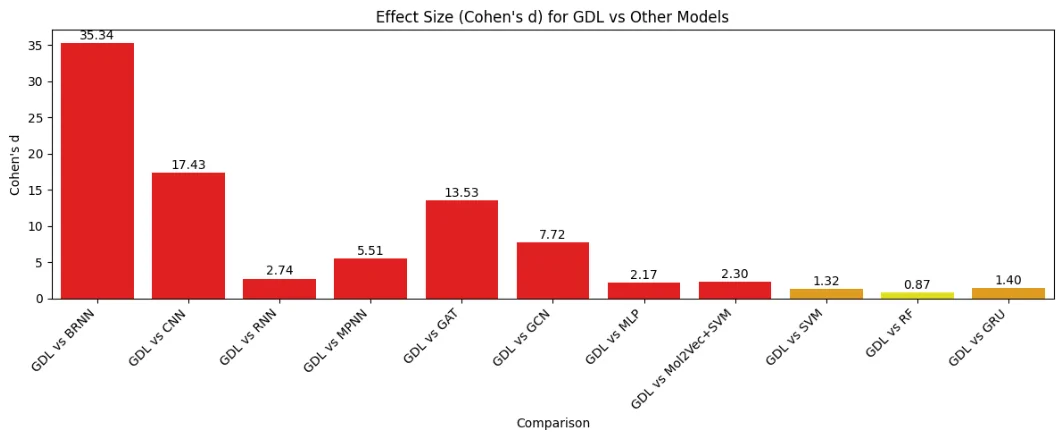
The graph shows the effect sizes (Cohen’s d) between the GDL model and other models.

In contrast, when comparing the GDL model with high-performance models such as SVM, RF, and GRU, the performance difference was found to be statistically insignificant (*p* > 0.05 after Holm correction). However, Cohen’s d effect sizes suggested relatively large practical differences between GDL and these models. Since these estimates were derived from only five paired cross-validation folds, they should be interpreted with appropriate caution. The corresponding effect size estimates are presented in Figure 7.

**Figure 8:**
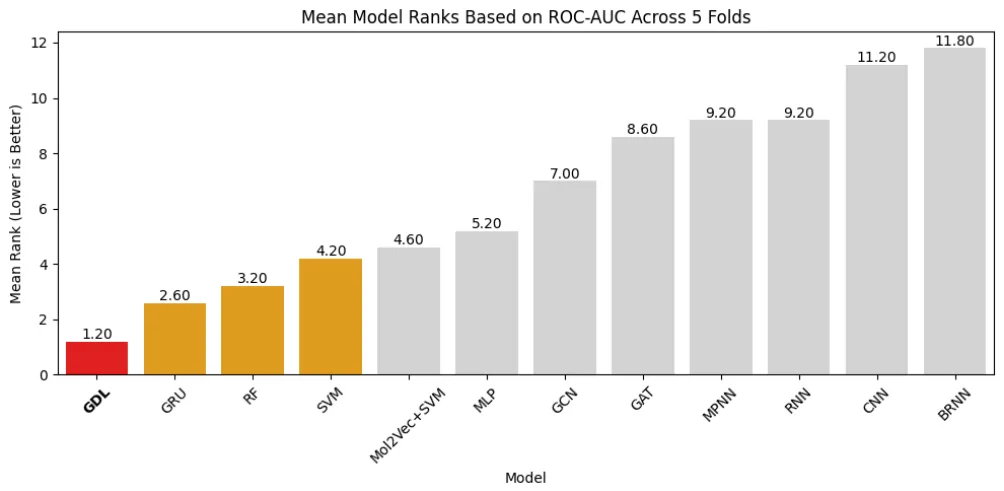
The graph shows the mean rank of the models based on 5-fold cross-validation results.

In addition, mean-rank analysis was performed to evaluate the overall performance ranking of the models, and the results are given in Figure 8. The results show that the GDL model has the lowest mean-rank value and exhibits the best overall performance compared to other models. Statistical analysis results reveal that the geometric deep learning approach provides a significant advantage over low and medium-performing models, but the differences among high-performing models are more limited and statistically less significant. This finding indicates that not only mean performance metrics but also criteria such as statistical significance and effect size should be considered together in model evaluation.

### 2.4. Highest-Ranked Compounds from the Independent Test Set and Predicted ADME Analysis

**Figure 9:**
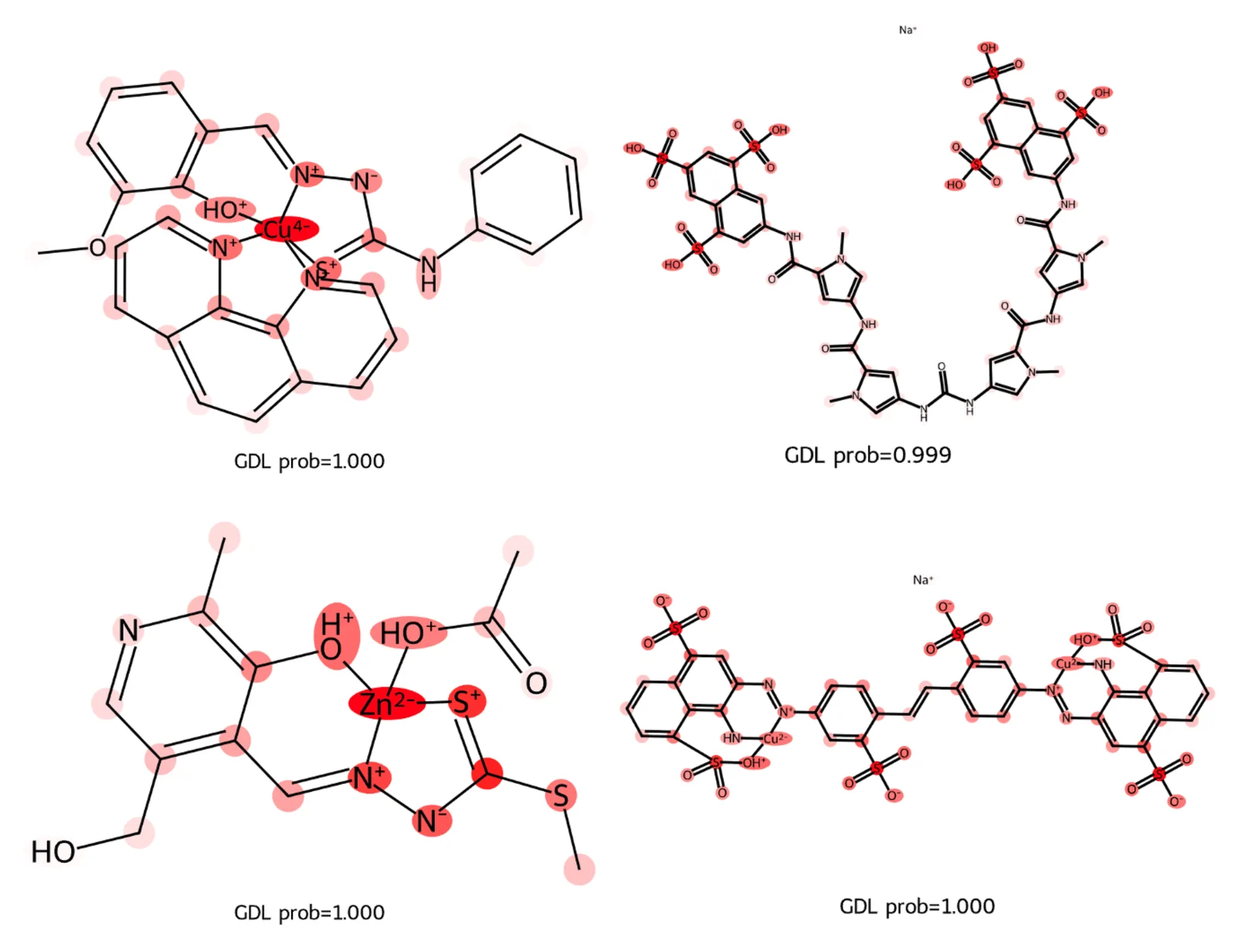
Visualizations showing the atomic and bond-level significance distributions for candidate molecules with high confidence scores for the GDL model.

**Figure 10:**
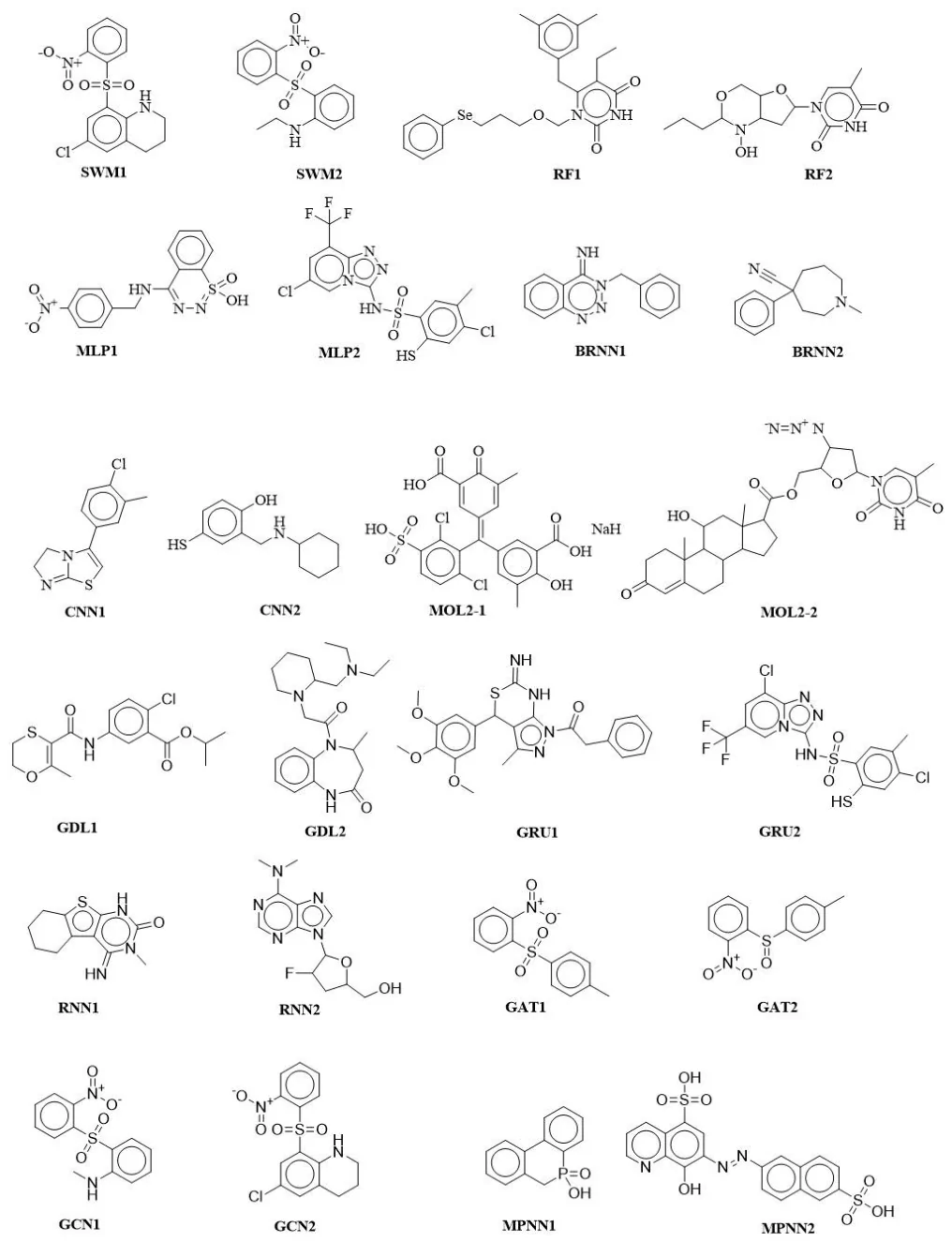
Chemical structures of compounds selected by methods.

In this section, a comprehensive evaluation was conducted on a total of 24 compounds, corresponding to the two highest-ranked compounds from the independent test set identified by each predictive model. The compounds were prioritized exclusively according to their predicted probabilities (*y_prob*) obtained from the independent test set; classification accuracy was not used for compound ranking. Subsequently, the prioritized compounds were characterized in terms of their predicted physicochemical properties (MW, LogP, HBD, HBA, TPSA, and rotatable bonds), predicted drug-likeness (Lipinski’s Rule of Five), and QED scores. In addition, for the GDL model that exhibits the highest discriminative performance in Figure 9, an interpretability analysis was conducted to better understand the model’s decision-making mechanism. The chemical structures of the selected candidate molecules are presented in Figure 10. In this context, visualizations showing atom- and bond-level importance scores were obtained for candidate molecules with high confidence scores (GDL prob ≈1.0). The results indicate that the model particularly focuses on chemically meaningful substructures such as aromatic rings, heteroatoms (N, O, S), and metal coordination centers (e.g., Cu^2+^ and Zn^2+^). These structures are known to play important roles in molecular binding, hydrogen bonding interactions, and electronic distribution, and are therefore associated with biological activity. This finding suggests that the GDL model does not merely learn statistical patterns, but is also able to capture chemically meaningful structure–activity patterns that are consistent with known molecular characteristics, in addition to achieving strong predictive performance.

SwissADME was used to predict the physicochemical and pharmacokinetic properties of a total of 24 compounds, corresponding to the two highest-ranked compounds from the independent test set identified by each of the 12 predictive models shown in Figure 10. The predicted properties included Lipinski’s Rule of Five compliance, gastrointestinal absorption, blood–brain barrier permeability (via the BOILED-Egg model), and drug-likeness scores. All predicted properties were comparatively evaluated to characterize the physicochemical and predicted pharmacokinetic profiles of the prioritized compounds [15,18,19]. When examining the prioritized compounds identified by the models in Figure 10, it is observed that different modeling approaches exhibit significant differences in the molecular characteristics of the prioritized compounds. Compounds prioritized by the GDL model (GDL1 and GDL2) are characterized by balanced molecular weight (MW ≈ 355–386), suitable lipophilicity (LogP ≈ 2.9–3.8), and high QED scores ( 0.81–0.83). These molecules also satisfy Lipinski’s rule of five and demonstrate high gastrointestinal absorption potential. Similarly, although some compounds prioritized by GRU and RF models exhibit high prediction probabilities (y_prob ≈1.0) and favorable ADME properties, in some cases violations of Lipinski’s rules or limitations in solubility and bioavailability were observed. In particular, highest-ranked compounds identified by the Mol2Vec+SVM pipeline were found to be weaker pharmacological candidates due to their high molecular weight (MW > 560), low QED scores (0.23), and multiple Lipinski rule violations. When sequence-based models (RNN, CNN, and BRNN) are examined, these models tend to identify molecules with lower molecular weights and simpler structural characteristics. However, although some of these molecules exhibit relatively high QED scores, their limited structural complexity may reduce their potential for strong biological activity.

**Figure 11:**
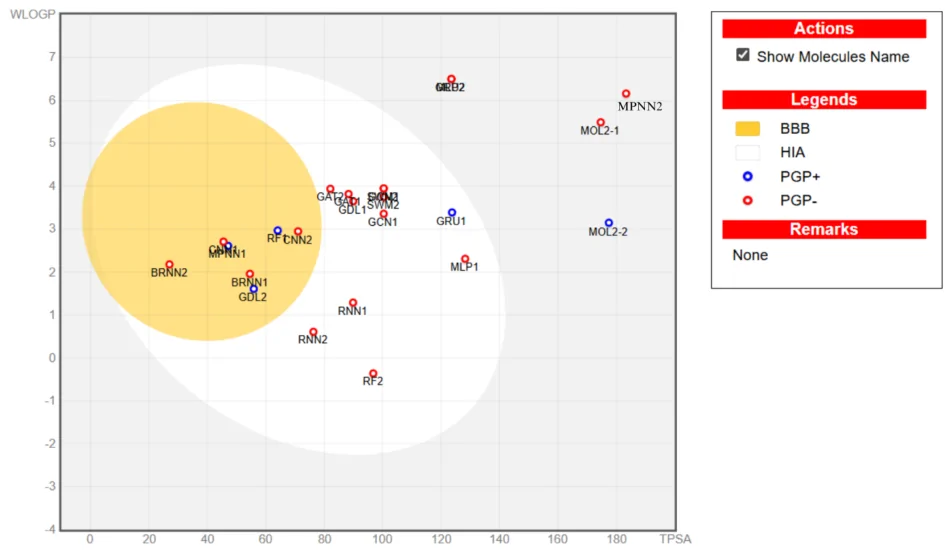
The BOILED-Egg model of all selected compounds.

Figure 11 illustrates the color distribution of the compounds based on their calculated values within the BOILED-Egg model. Localization within the yellow region suggests a high probability of blood–brain barrier permeability, whereas positioning in the white region indicates a likelihood of gastrointestinal absorption via passive diffusion. Compounds that fall outside both regions are considered unlikely to exhibit either of these properties. When the results of the compounds selected by the methods were examined through the program, it was observed that the compounds selected by the BRNN, MPNN, GDL, RF, and CNN methods were in the yellow region, indicating that they could cross the blood–brain barrier, while the compounds selected by the Mol2Vec method were completely outside the yellow region (Figure 11).

**Figure 12:**
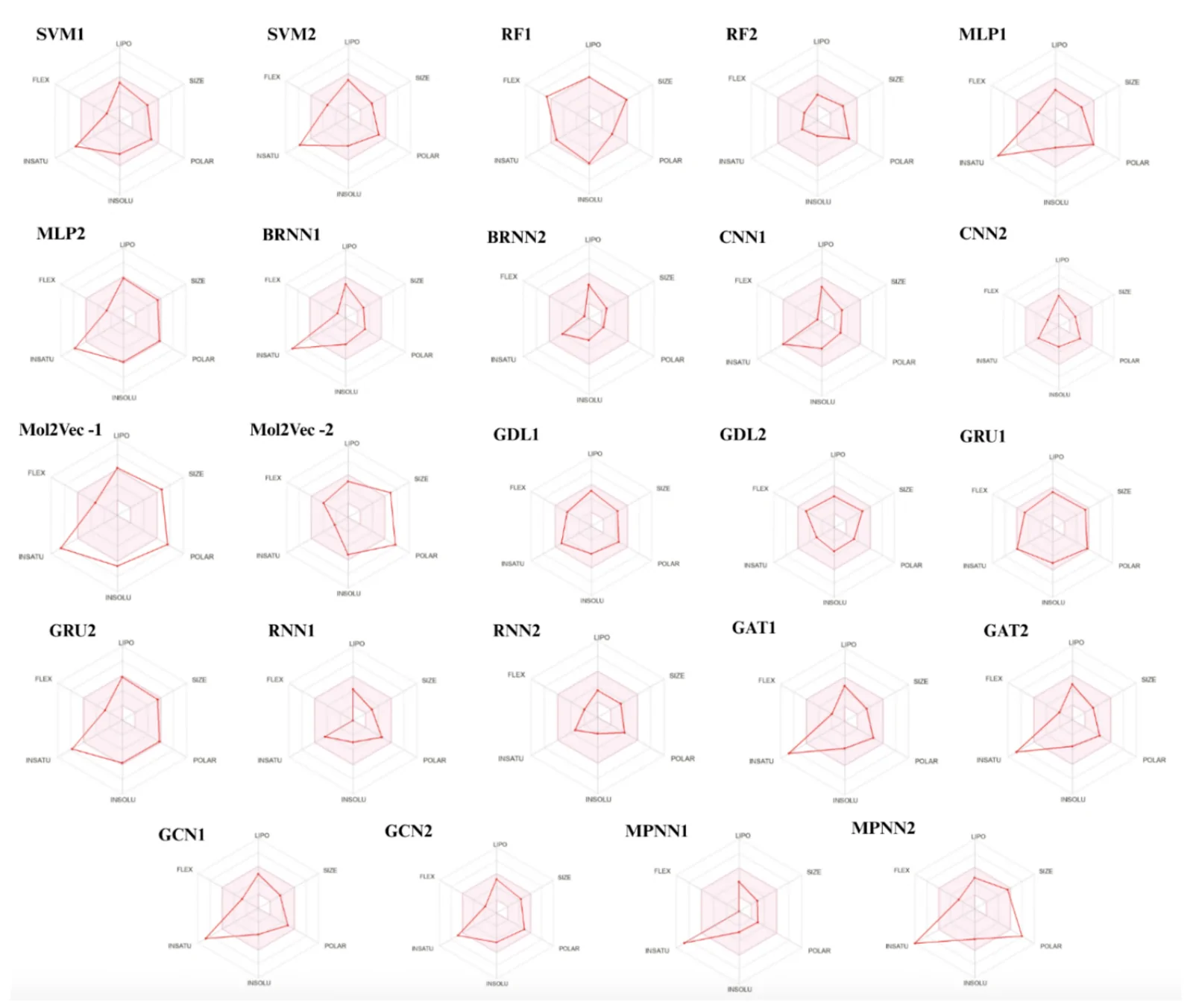
Predicted physicochemical space for oral bioavailability.

The abbreviations presented in Figure 12 are defined in the accompanying table as follows: Lipo (lipophilicity) corresponds to XLOGP3; Flex (flexibility) represents the number of rotatable bonds; Size (molecular weight) is indicated by MW; Insatu (insaturation) refers to the Fraction Csp3; Insolu (solubility) is expressed as LogS; and Polar (polarity) corresponds to TPSA. While the compounds selected by the SVM, BRNN, CNN, GDL, RNN, GAT, and GCN methods fully complied with the Lipinski, Ghose, Veber, Egan, and Muegge rules, which indicate drug-like properties, the compounds selected by the RF, MLP, Mol2Vec, GRU, and MPNN methods did not comply with some of these rules [17,39,40,41]. When the LogS values were examined, it was observed that the solubility of all compounds ranged from −2.06 to −6.61, and their dissolution rates were between poorly and soluble. When the bioavailability score (F) was examined, it could be said that the compounds selected by the Mol2Vec+SVM pipeline and MPNN methods were lower than those selected by the other methods. The cLogP values, which represent the lipophilicity of the compounds, ranged from 0.65 to 4.16. When evaluated within themselves, the GAT1-2 compounds were found to be compatible with each other (Table 9 and Table 10).

Molecular docking studies were conducted to evaluate the interaction potential of the GDL1 compound, identified by the graph-based GDL model as a high-ranking candidate compound, against three major HIV-1 therapeutic targets: HIV-1 integrase, HIV-1 protease, and HIV-1 reverse transcriptase (RT). Docking calculations were performed using ChemOffice Ultra 8.0.3 (PerkinElmer Inc.), while docking simulations were carried out with AutoDock Vina (version 1.1.2) implemented in AutoDock (version 1.5.7) [42,43]. The crystal structures of HIV-1 protease (PDB ID: 1AJV), HIV-1 integrase (PDB ID: 1QS4), and HIV-1 reverse transcriptase (PDB ID: 2ZD1) were retrieved from the Protein Data Bank (PDB) and prepared for docking analyses [44,45,46]. Docking protocol validation was performed using the Lamarckian Genetic Algorithm together with the AutoDock4.2 scoring function [47]. During protein preparation, water and heteroatoms outside a significant 6 Å distance from the co-crystal ligand of each protein were removed and replaced with polar hydrogens. Grid boxes are positioned around the co-crystal ligands for all receptors and their sizes are set as Grid Points =40×40×40. The number of genetic algorithm clamping runs was 50 for each study. For AutoDock Vina, the exhaustiveness settings were not changed; the calculation was performed using the standard, default values. The docking protocol was supported by reference root mean square deviation (RMSD) values of 0.96 for AHA006 (PDB ID: NMB) with HIV-1 Protease (1AJV), 2.41 for 5CITEP (PDB ID: 100) with HIV-1 Integrase (1QS4), and 0.58 for Rilpivirine (PDB ID: T27) with HIV-1 Reverse Transcriptase (2ZD1), consistent with our previous study [16]. Protein–ligand interactions were analyzed and visualized using Maestro Release 2026-1 (version 14.7) [48].

The highest-ranked compound identified by the graph-based GDL model (GDL1) was further evaluated using molecular docking against three major HIV-1 therapeutic targets, namely HIV-1 integrase, HIV-1 protease, and HIV-1 reverse transcriptase (RT). Molecular docking provides computational insight into the potential interactions between small molecules and target proteins and serves as a complementary in silico approach for candidate prioritization. The docking results suggested favorable binding affinities for the GDL1 compound toward all three HIV-1 targets, with predicted inhibition constants in the micromolar (μM) range for HIV-1 integrase and in the nanomolar (nM) range for HIV-1 protease and HIV-1 reverse transcriptase. These computational findings indicate the potential for target binding but should not be interpreted as confirmation of inhibitory activity or therapeutic efficacy. Experimental validation remains essential to determine the biological activity, safety, and therapeutic potential of the prioritized compound. The highest-ranked compound identified by the graph-based GDL model (GDL1) was selected for molecular docking based on its favorable predicted ADME profile and QED score. As a complementary in silico analysis, its predicted interactions with HIV-1 integrase, HIV-1 protease, and HIV-1 reverse transcriptase were evaluated using AutoDock4 and AutoDock Vina. The molecular docking results obtained using AutoDock4 and AutoDock Vina are summarized in Table 11.

Consequently, the molecular docking analysis provided complementary computational information regarding the predicted binding profile of the GDL1 compound selected by the GDL model. To further examine its predicted binding characteristics, docking protocol validation was performed using the co-crystallized ligands of HIV-1 integrase (PDB ID: 1QS4; ligand ID: 100), HIV-1 protease (PDB ID: 1AJV; ligand ID: NMB), and HIV-1 reverse transcriptase (PDB ID: 2ZD1; ligand ID: T27, rilpivirine). The binding interactions of GDL1 were subsequently compared with those of the corresponding co-crystallized ligands. Similar interaction patterns with several key amino acid residues were observed, suggesting that GDL1 occupies comparable regions within the binding pockets.

**Figure 13:**
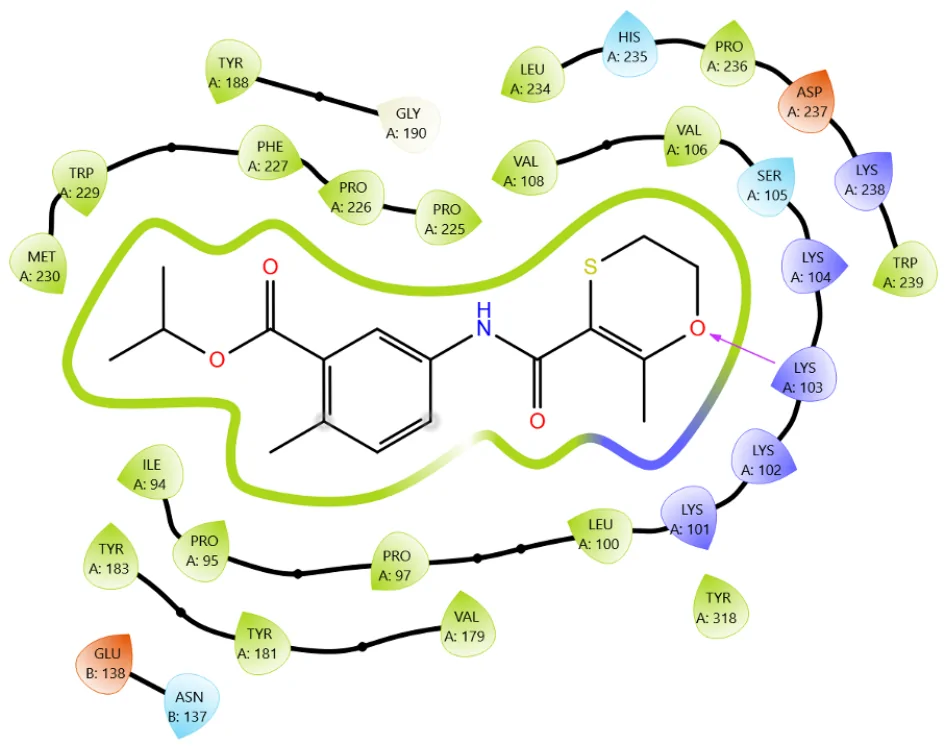
Two-dimensional binding state, interacting residues of HIV-1 reverse transcriptase with GDL1.

**Figure 14:**
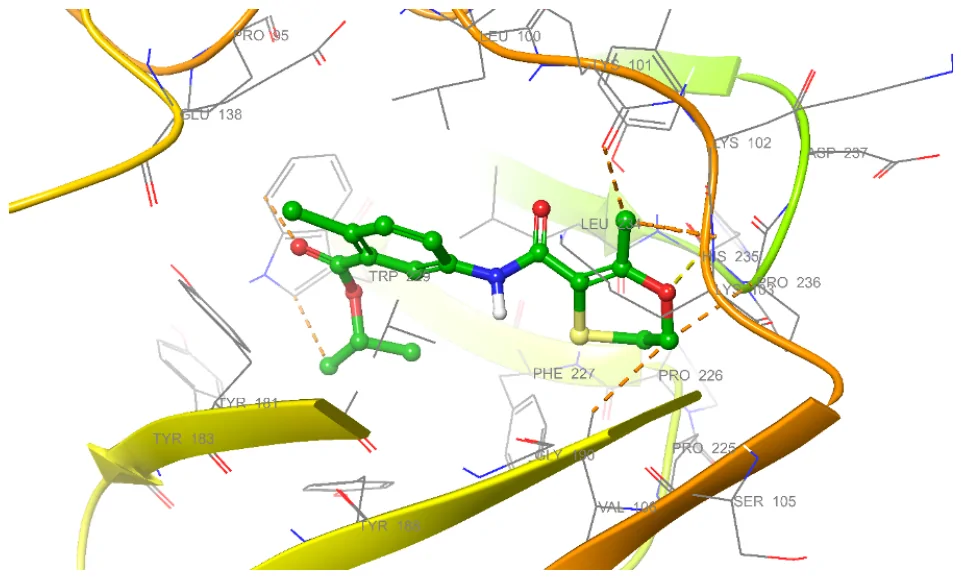
Three-dimensional binding state, interacting residues of HIV-1 reverse transcriptase with GDL1.

Among the three investigated targets, HIV-1 reverse transcriptase exhibited the most favorable predicted binding affinity, with an estimated inhibition constant ($K_{i}$) of 163.49 nM and an AutoDock4 docking score of −9.26 kcal/mol. The predicted binding mode of GDL1 and its interactions with the active-site residues of HIV-1 reverse transcriptase are illustrated in Figure 13 and Figure 14.

### 2.5. Discussion

This study presents a comprehensive evaluation of machine learning (ML), deep learning (DL), and graph-based (GDL) approaches for predicting the biological activity of HIV-related compounds. It distinguishes itself from traditional performance comparisons by integrating statistical validation with complementary predicted ADME and drug-likeness characterization. Regarding predictive performance, the results detailed in Section 2.1 demonstrate that the graph-based GDL model achieved the highest ROC–AUC values, exhibiting a superior ability to capture complex structural relationships within molecular graphs. This observation is consistent with recent literature highlighting the effectiveness of graph-based architectures, particularly Message Passing Neural Networks (MPNNs), in molecular property prediction tasks [25]. Although the graph-based GDL model achieved the highest ROC–AUC (0.956) and precision (0.997) among all evaluated models, its comparatively lower recall (0.748) indicates a more conservative prediction behavior, implying that approximately one-quarter of the experimentally active compounds may remain unidentified. Such behavior may be less desirable during the early stages of virtual screening, where maximizing the recovery of active compounds is often prioritized. Conversely, the high precision of the GDL model substantially reduces the number of false-positive predictions, thereby improving the reliability of the highest-ranked compounds selected for downstream computational analyses such as ADME evaluation and molecular docking. Therefore, the choice of predictive model should depend on the intended application. Models with higher recall may be preferable for broad primary screening, whereas models with higher precision may be more suitable for prioritizing a smaller set of high-confidence compounds for subsequent computational or experimental investigation. These findings emphasize that model selection should be based on the trade-off between precision and recall rather than on a single performance metric. However, in contrast to previous studies that primarily focused on predictive performance, this study further investigates whether these performance gains are accompanied by differences in the predicted physicochemical, ADME, and drug-likeness characteristics of the highest-ranked compounds from the independent test set [23,25,49,50]. The statistical analysis presented in Section 2.3 further confirms that the performance differences between the models are not coincidental but statistically significant. While Friedman test results show a global difference between the models, subsequent comparisons reveal that GDL performs significantly better than most underlying models. However, the lack of statistical significance between GDL, GRU, RF, and SVM indicates that, despite differences in the underlying architectures, the high-performance models converge in terms of predictive ability. Furthermore, the simple RNN exhibited substantially higher variability across the five cross-validation folds than the remaining models. Although the present study did not investigate the underlying optimization dynamics, this variability suggests that the baseline RNN architecture was less stable than more advanced recurrent architectures such as GRU under the predefined experimental setting. Since all models were evaluated using the same experimental protocol, the observed variability should be interpreted as a characteristic of the baseline RNN implementation rather than evidence of a methodological issue. Nevertheless, this higher variability did not alter the overall comparative conclusions of the present study because all evaluated models were trained and assessed using identical data partitions, the same five-fold cross-validation protocol, and the same evaluation metrics. Future work may investigate alternative hyperparameter configurations, weight initialization strategies, and architecture-specific training procedures to further improve the stability of simple recurrent networks. The additional sensitivity analysis further demonstrated that the predictive performance of the Random Forest classifier remained consistent when the inactive subset compounds were replaced by chemically comparable alternatives. This finding indicates that the conclusions of the present study are not driven by a particular inactive subset but are reproducible under chemically constrained sampling conditions. A significant contribution of this study emerges when relating predictive performance to ADME characteristics. The subsequent molecular docking analysis was performed only for the highest-ranked GDL1 compound and therefore should be interpreted as an independent complementary evaluation rather than as evidence of a correlation between predicted ADME properties and docking performance. As illustrated in Table 9 and Table 10, and further corroborated by the BOILED-Egg analysis (Figure 11), models such as GDL, RF, RNN, and CNN tend to generate molecules that adhere to drug-likeness rules and exhibit favorable pharmacokinetic profiles (Figure 12). In contrast, certain models (e.g., Mol2Vec+SVM and some MPNN-based models) prioritize compounds that, despite high predicted probabilities, exhibit multiple Lipinski rule violations or less favorable predicted ADME and drug-likeness profiles. This observation highlights an important limitation of many existing studies, where model performance is evaluated solely using classification metrics without considering the predicted physicochemical, ADME, and drug-likeness characteristics of the highest-ranked compounds from the independent test set. More specifically, the superior performance of the graph-based GDL model can be attributed to its ability to encode both local and global structural dependencies within molecular graphs through message-passing mechanisms. This enables the model to learn expressive molecular representations that more effectively capture structural relationships relevant to biological activity prediction. In contrast, sequence-based models such as RNNs and CNNs primarily rely on SMILES representations, which may not fully capture topological and relational information, potentially leading to the prioritization of compounds with comparatively simpler structural characteristics. Another key observation is that a high predicted probability ($y_{prob}$) does not necessarily correspond to favorable predicted physicochemical, ADME, and drug-likeness characteristics. For instance, some models assigned near-perfect prediction probabilities to compounds that exhibited high molecular weights, excessive polarity, or violations of Lipinski’s Rule of Five. This finding reinforces the importance of complementing predictive performance evaluation with predicted physicochemical, ADME, and drug-likeness characterization.

Compared to existing literature, including recent MPNN-based frameworks focused on improving predictive performance and interpretability through explainability modules, this study provides a more comprehensive evaluation framework. Specifically, it integrates multi-model comparative analysis, rigorous statistical validation, and complementary predicted physicochemical, ADME, and drug-likeness characterization of the highest-ranked compounds from the independent test set. This multi-layered approach enables a more comprehensive assessment of predictive performance together with the predicted molecular characteristics of the prioritized compounds. Recent AI-focused studies on HIV have generally concentrated on prediction accuracy, explainability, multi-target screening, or de novo molecular generation [23,25,49,50,51]. In contrast, the present study links predictive performance with the predicted physicochemical, ADME, and drug-likeness characteristics of the highest-ranked compounds from the independent test set by unifying multi-model comparative analysis, post hoc statistical significance testing, and complementary predicted ADME characterization within a single evaluation framework. Graph-based approaches have recently gained prominence in HIV-related molecular prediction, as they encode atom–bond topology more accurately than sequence-based representations alone. This explains why our GDL model achieved the highest overall ranking and competitive performance against most baseline models. However, our ADME results further demonstrate that predictive superiority does not automatically guarantee pharmacologically superior outcomes. This distinction is often under-explored in recent HIV-focused AI studies, where classification or generative success is reported without a systematic cross-model ADME comparison [5,38,52]. The findings suggest that the most effective models are not necessarily those with the highest predictive performance, but rather those that combine strong predictive performance with favorable predicted physicochemical, ADME, and drug-likeness characteristics. Accordingly, comparative evaluation of predictive performance together with predicted molecular characteristics provides a more comprehensive basis for assessing the highest-ranked compounds identified by different models. Although the present study employed an independent hold-out test set together with five-fold cross-validation during model development, external or temporal validation using an independently collected HIV dataset would provide an additional assessment of model generalizability. However, publicly available HIV bioactivity datasets with comparable assay conditions and molecular annotations remain limited. Consequently, external validation was the scope of the present study. Future work will investigate the proposed framework using newly curated external datasets as they become available. Similar recommendations have also been discussed in previous ligand-based virtual screening studies [53]. As a limitation of the study, the present study focused on the comparative evaluation of machine learning and deep learning models under a unified experimental protocol. Therefore, only the two highest-ranked compounds per model were selected as representative examples for comparative ADME characterization. Although this approach enabled a consistent comparison across all evaluated models, the assessment of a larger, pre-specified subset of top-ranked compounds could provide a more comprehensive characterization of the models’ prediction behavior. Such an expanded analysis, including systematic ADME evaluation of a larger candidate set and prospective external validation, will be considered in future studies. Furthermore, the present study was designed to compare different machine learning, deep learning, and graph-based models under a unified experimental protocol rather than to evaluate robustness under highly imbalanced screening conditions. To minimize the confounding influence of extreme class imbalance on comparative model evaluation, all models were trained and evaluated using the same curated dataset. The presented findings should be interpreted as a comparative assessment under controlled experimental conditions rather than as evidence of model performance in large-scale real-world virtual screening campaigns involving highly imbalanced datasets. Future studies will evaluate the proposed framework using the complete MoleculeNet HIV dataset together with additional highly imbalanced screening datasets to further investigate model robustness under realistic class distributions.

In addition, since the MoleculeNet HIV benchmark is derived from cell-based antiviral screening assays, it is important to acknowledge that measured antiviral activity may, in some cases, be influenced by nonspecific cellular effects rather than target-specific antiviral mechanisms. Drug-induced phospholipidosis has been reported as a potential confounding factor in antiviral screening campaigns for several viral systems [54]. However, to the best of our knowledge, its relevance to the MoleculeNet HIV dataset has not been systematically established. Therefore, the computational predictions presented in this study should be interpreted as a prioritization framework rather than evidence of confirmed biological activity. Future studies may incorporate complementary phospholipidosis prediction tools, such as the AMALPHI platform, to further refine candidate selection prior to experimental validation [55]. Although molecular docking provides complementary evidence regarding potential protein–ligand interactions, neither docking nor ADME predictions can replace experimental validation. Future studies will therefore include in vitro antiviral activity and cytotoxicity assays to evaluate the biological activity and safety of the prioritized compounds.

## 3. Materials and Methods

**Figure 15:**
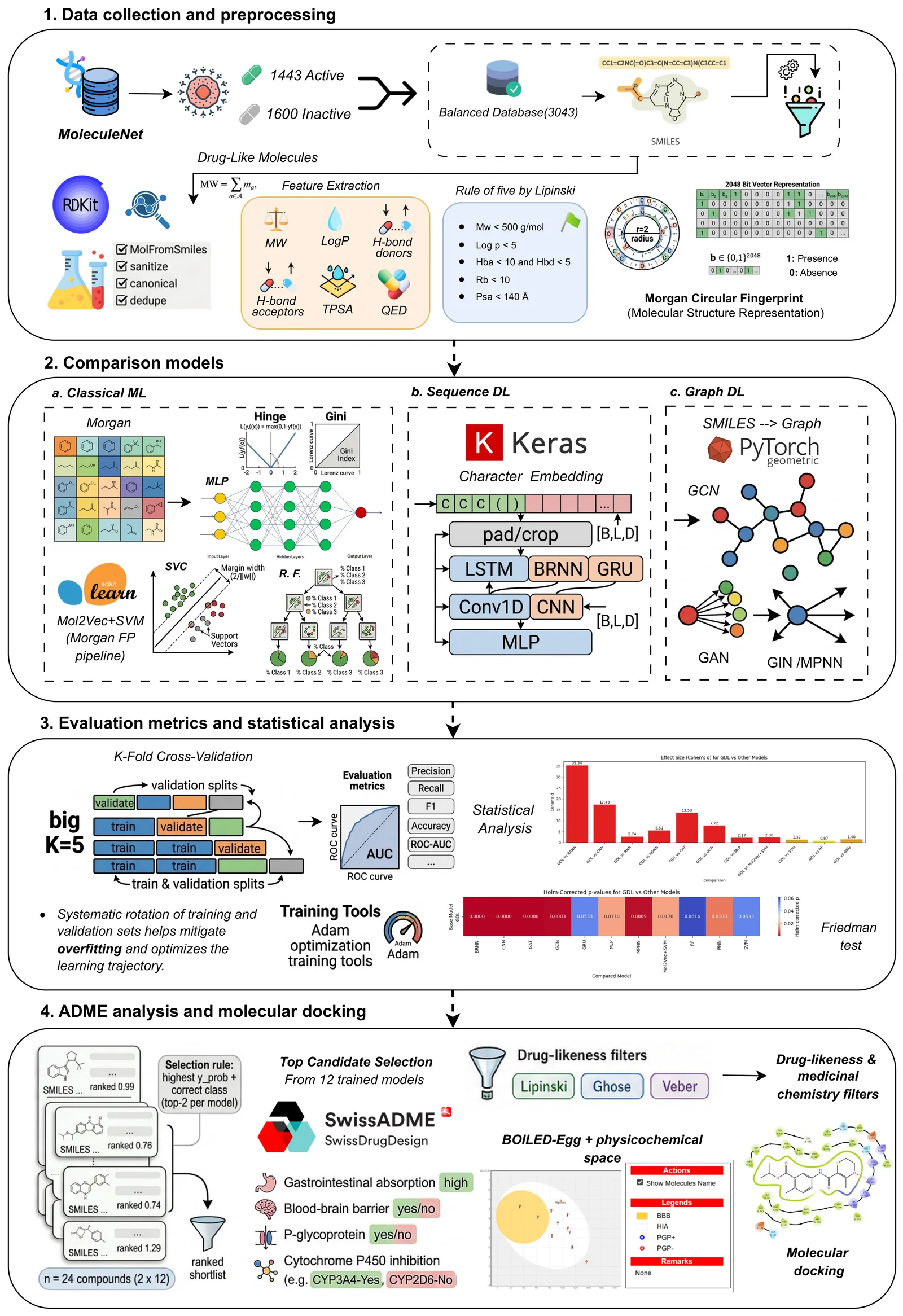
Workflow of the study for comparative evaluation of different models in HIV molecular bioactivity prediction.

This study develops an integrated experimental framework encompassing machine learning (ML), deep learning (DL), and graph-based geometric deep learning (GDL) approaches for the HIV bioactivity prediction problem. The proposed methodology presents a multi-layered structure covering the inference of molecular representations, model training, and evaluation processes, starting from data collection and preprocessing stages. As illustrated in Figure 15, the experimental pipeline consists of data preparation, model development, comparative performance evaluation, statistical analysis, and complementary predicted ADME characterization. First, data collection and preprocessing procedures are described, including dataset features, cleaning steps, and data splitting strategies. Then, molecular descriptors and drug-likeness assessment criteria are introduced, together with the physicochemical properties and filtering rules used to evaluate the predicted pharmacokinetic and drug-likeness characteristics of the highest-ranked compounds from the independent test set. Subsequently, the machine learning, deep learning, and geometric deep learning models used in this study are described in detail, along with their architectural configurations, training strategies, and hyperparameter settings. All models were trained under the same data splitting strategy and the same 5-fold cross-validation protocol to ensure a fair and comparable experimental environment. Hyperparameter configurations were selected based on commonly adopted settings reported in the literature together with preliminary experiments conducted on the training data, rather than through a unified automated hyperparameter optimization procedure. Furthermore, model performance is evaluated not only using classification metrics but also through statistical analyses and complementary predicted ADME and drug-likeness assessments. The overall workflow of the proposed experimental framework is presented in Figure 15. Through this comprehensive framework, the relationship between predictive performance and the predicted pharmacokinetic and drug-likeness characteristics of prioritized compounds has been systematically investigated. To promote transparency and reproducibility, the processed dataset, model implementations, software environment information, and outputs have been made publicly available in a GitHub repository: https://github.com/aycabostancioglu/HIV-ADME-Framework (accessed on 9 June 2026).

### 3.1. Data Collection and Preprocessing

The dataset used in this study was obtained from MoleculeNet benchmarking platform, a widely accepted resource for molecular machine learning research. Specifically, the HIV dataset, which includes experimentally measured inhibition data related to HIV replication, was used. The original dataset consists of 41,127 molecules, 1443 of which show anti-HIV activity and 39,684 are inactive. However, due to the highly unbalanced distribution and the presence of ambiguous or weak activity descriptions, a curated subset was constructed to ensure reliable learning of molecular activity models. Therefore, molecules with clearly defined and experimentally validated activity labels were selected, resulting in a refined dataset of 3043 unique compounds, 1443 of which are active and 1600 are inactive. The reduced HIV dataset used in this study was constructed during the initial stage of the TÜBİTAK-funded project through expert curation of the MoleculeNet HIV dataset. The active compounds were retained, whereas the inactive compounds were selected after excluding ambiguous activity annotations and retaining compounds with clearly defined, experimentally validated inactive labels. This curated subset provides a more consistent and less noisy representation of the structure–activity relationship, thereby facilitating more reliable comparative evaluation of predictive models for HIV bioactivity. The dataset used in this study was obtained from MoleculeNet benchmarking platform, a widely accepted resource for molecular machine learning research. Specifically, the HIV dataset, which includes experimentally measured inhibition data related to HIV replication, was used. The original dataset consists of 41,127 molecules, 1443 of which show anti-HIV activity and 39,684 are inactive. However, due to the highly unbalanced distribution and the presence of ambiguous or weak activity descriptions, a compiled subset was created to ensure reliable learning of molecular activity models. Therefore, molecules with clearly defined and experimentally validated activity labels were selected, resulting in a refined dataset of 3043 unique compounds, 1443 of which are active and 1600 are inactive. This subset provides a more consistent and noise-free representation of the structure-activity relationship, enabling more robust modeling of HIV biological activity. All molecules were represented using the SMILES (Simplified Molecular Input Line Entry System) format. To ensure structural consistency, all SMILES strings were processed using the RDKit cheminformatics toolkit, and each molecule was converted into a canonical molecular representation [13]. Invalid SMILES entries and duplicate molecules were removed during preprocessing to improve data quality. Following standard cheminformatics procedures, a series of physicochemical descriptors were calculated for each molecule, including molecular weight MW, logarithm of the partition coefficient LogP, number of hydrogen bond donors HBD, number of hydrogen bond acceptors HBA, topological polar surface area TPSA, number of rotatable bonds, and quantitative estimation of drug-likeness QED. These descriptors provide a quantitative representation of the molecular structure and are commonly used in activity prediction tasks. Molecular weight is expressed by summing the atomic masses (Equation (1)). Lipinski’s Five Rules give the commonly used upper thresholds for oral bioavailability collectively in Equation (2). In this study, the *Lipinski-pass* tag is assigned according to whether these four inequalities are met simultaneously. TPSA and number of rotatable bonds summarize the balance between polarity and flexibility, QED provides a single “drug-likeness” score derived from the combination of these properties. These descriptors not only serve as model inputs but are also used to convey the chemical profile of the sample compounds to the reader in the results tables (e.g., MW–QED columns in the SVM output table).

(1) $$\mathrm{MW}=\sum_{a\in A}m_{a},$$

where $m_{a}$ is the mass of the relevant atom and A the cluster of atoms in the molecule.

(2) $$\begin{array}{cc} \; & \mathrm{MW}\leq 500,\;\mathrm{LogP}\leq 5,\\ \; & N_{\mathrm{HBD}}\leq 5,\;N_{\mathrm{HBA}}\leq 10. \end{array}$$

**Figure 16:**
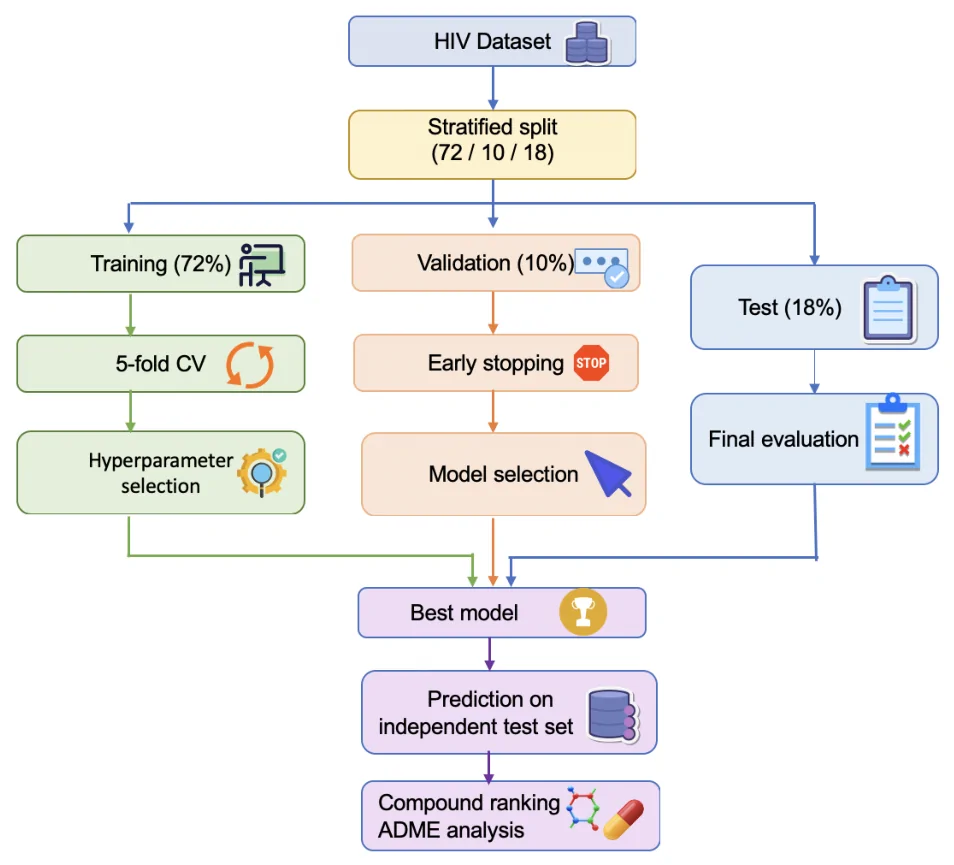
Data partitioning and model development workflow.

The complete HIV dataset was initially divided into three mutually exclusive subsets using stratified sampling: 72% for training, 10% for validation, and 18% for independent testing. The independent test set was isolated before model development and was not used during model training, hyperparameter selection, early stopping, or model selection. Model development was performed exclusively using the training subset. Five-fold cross-validation was employed to estimate model performance during model development. Hyperparameter values were selected based on preliminary experiments and commonly adopted settings reported in the literature rather than through a unified automated hyperparameter optimization procedure. For deep learning models, the validation subset was used solely for early stopping and model selection, whereas for machine learning models it was used only to verify the selected hyperparameter configuration. After model development was completed, the final model was evaluated once using the independent test set. The prioritization of the highest-ranked compounds from the independent test set and all subsequent predicted ADME and drug-likeness analyses were performed exclusively. Furthermore, any resampling or inactive-subset replacement procedures introduced during the sensitivity analysis were restricted to the training subset only. Neither the validation set nor the independent test set was modified at any stage of model development or performance evaluation. Figure 16 shows the experimental workflow summarizing the data partitioning strategy and model development protocol.

Following model evaluation, two compounds were selected from each predictive model for comparative physicochemical, ADME, and drug-likeness characterization. Compound selection was performed exclusively using the independent hold-out test partition and was based solely on the predicted probability ($y_{prob}$). Model-level performance metrics, such as classification accuracy, were not used for ranking individual compounds. For each predictive model, stratified five-fold cross-validation was first performed to estimate predictive performance. The cross-validation fold achieving the highest test ROC–AUC value was identified, and the trained model corresponding to that fold was used to generate predictions for the independent test partition of the same fold. No aggregation of predicted probabilities across cross-validation folds was performed. Compounds within the independent test partition were ranked according to their predicted probabilities, and the two compounds with the highest $y_{prob}$ values were selected for subsequent comparative analysis. For each selected compound, the predictive model, cross-validation fold, true experimental label, predicted class, and predicted probability were recorded. This procedure provides a transparent and fully documented compound prioritization protocol, enabling the compound selection process to be reproduced. The complete compound selection protocol is summarized in Table 12.

### 3.2. Classical Machine Learning Models

In this study, a set of classical machine learning models was employed as baseline approaches to evaluate the effectiveness of advanced deep learning and graph-based geometric deep learning methods in predicting molecular bioactivity. These models include Support Vector Machine (SVM), Random Forest (RF), Multi-Layer Perceptron (MLP), and a Mol2Vec+SVM pipeline. The inclusion of these methods provides a robust comparative framework, enabling a comprehensive assessment of model performance across different representation paradigms. To ensure a fair comparison, RF, SVM, and MLP were trained using molecular fingerprint representations, whereas the Mol2Vec+SVM pipeline employed continuous molecular embeddings generated by the Mol2Vec algorithm. For the fingerprint-based models (RF, SVM, and MLP), Extended-Connectivity Fingerprints (ECFP), also known as Morgan fingerprints, were generated from SMILES representations using the RDKit library. These fingerprints encode substructural information of molecules into fixed-length binary vectors, allowing traditional machine learning algorithms to effectively process chemical structures. For the fingerprint-based classical models, each compound is encoded with a circular fingerprint (*r* = 2, 2048 bits; the bit vector b is symbolized in Equation (3)). SVM, learns a segregating hyperplane under the kernel RBF decision function and kernel definition are given in Equation (4). RF, is a combination of multiple decision trees; the split at each node is chosen by minimizing the Gini impurity as in Equation (5). Hyperparameter details are listed in Table 13. Hyperparameter values were selected according to literature recommendations and preliminary experiments on the training data.

The SVM model was implemented using a radial basis function (RBF) kernel, which is well-suited for capturing non-linear relationships in high-dimensional feature spaces. Hyperparameters such as the regularization parameter (C) and kernel coefficient (γ) were selected to balance model complexity and generalization performance. Random Forest, an ensemble-based method, was utilized to leverage multiple decision trees trained on different subsets of the data, thereby improving robustness and reducing overfitting. The number of trees and maximum depth were carefully selected to ensure stable performance across folds. The MLP model represents a shallow neural network baseline, consisting of fully connected layers with non-linear activation functions. Although simpler than deep architectures, MLP provides a useful benchmark for evaluating the added value of more complex deep learning models. In addition, a Mol2Vec+SVM pipeline was incorporated to capture semantic relationships between molecular substructures. In this hybrid setup, molecular embeddings generated via Mol2Vec were used as input features to an SVM classifier, enabling a comparison between handcrafted fingerprints and learned molecular representations. All classical models were trained and evaluated under the same cross-validation protocol to ensure consistency and comparability of results.

(3) $$\begin{array}{cc} b & \in {\left\{0,1\right\}}^{2048},\\ b_{k} & =1\left[k\mathrm{The}\;\mathrm{underlying}\;\mathrm{structure}\;\mathrm{is}\;\mathrm{present}\;\mathrm{in}\;\mathrm{the}\;\mathrm{molecule}\right]. \end{array}$$

(4) $$\begin{array}{cc} f(x) & =\mathrm{sign}\left(\sum_{i\in S\;V}\alpha_{i}y_{i}K\left(x_{i},x\right)+b\right),\\ K(x,x^{\prime }) & =\exp\left(-\gamma \;∥x-x^{\prime }{∥}_{2}^{2}\right). \end{array}$$

(5) $$I_{G}\left(D\right)=1-\sum_{k=1}^{C}p_{k}^{2},$$

Here $p_{k}$, the class *k* at the node represents the sample rate and *C* represents the number of classes. The next subsection addresses the comparative bug defined under a different filename with the same general SVM kernel.

In addition to fingerprint-based representations, a Mol2Vec + SVM pipeline was also employed to incorporate distributional semantics of molecular substructures into the learning process. Mol2Vec is an unsupervised representation learning technique inspired by natural language processing, where molecular fragments are treated analogously to words and embedded into a continuous vector space. This approach enables the capture of contextual relationships between substructures, which are not explicitly represented in traditional fingerprint methods. In the Mol2Vec + SVM pipeline, molecules are first decomposed into substructure fragments, which are then embedded into fixed-length vector representations. These embeddings are subsequently used as input features for an SVM classifier, allowing the model to learn non-linear decision boundaries in the learned feature space. By combining representation learning with a classification algorithm, this hybrid approach aims to enhance the modeling of structure–activity relationships compared to purely handcrafted descriptors.

### 3.3. Sequence-Based Deep Learning Models

The SMILES strings were first embedded at the character level, then RNN, Bidirectional RNN (BRNN), GRU, and CNN were used to model sequential dependencies. The RNN hidden update is given in Equation (6), the GRU gate updates are given in Equation (7). MLP serves as a fully connected neural network baseline for comparison, whereas CNN with Conv1D layers is employed as a feature extractor to capture local patterns in sequential data [56]. In the sequence models, the SMILES characters were first converted to integer IDs (embedding), then padded or clipped to a fixed length, allowing molecules of different lengths to fit into the same tensor shape. Repetitive structures are particularly suitable for capturing patterns sensitive to the order of sub-sequences; The ‘CNN’ layers highlight local ‘*n*-gram’-like structures. ‘BRNN’ attempts to balance long-range dependencies that might be missed in a one-way ‘RNN’ by collecting context both forward and backward (Table 13).

(6) $$h_{t}=\phi \;\left(W_{xh}x_{t}+W_{hh}h_{t-1}+b_{h}\right).$$

(7) $$\begin{array}{cc} z_{t} & =\sigma \;\left(W_{z}\left[h_{t-1}⊕x_{t}\right]\right),\\ r_{t} & =\sigma \;\left(W_{r}\left[h_{t-1}⊕x_{t}\right]\right),\\ {\tilde{h}}_{t} & =\tanh\;\left(W_{h}\left[\left(r_{t}⊙h_{t-1}\right)⊕x_{t}\right]\right),\\ h_{t} & =\left(1-z_{t}\right)⊙h_{t-1}+z_{t}⊙{\tilde{h}}_{t}. \end{array}$$

The updates above mathematically fix the formal difference between the simple repetitive structure and the more stable gate mechanism GRU, respectively. The representations learned from the SMILES sequence, enriched with repetitive and convolutional layers, provide a sequence-oriented point of contrast for the transition to graph-based representations, which will be discussed in the next subsection, with the resulting chemical molecule diagrams.

### 3.4. Graph-Based Models

Molecules are represented as graphs G=(V,E) derived from SMILES, with atoms as nodes and chemical bonds as edges, consistent with the molecular graph representation used in [57]. GCN is performed with the normalized proximity matrix $\tilde{A}$ as in Equation (8). In the message transmission/GIN line, neighbor representations are summed as in Equation (10) and combined with node updates. The graph-based GDL model is implemented using GIN-style message-passing layers within this framework. The proposed graph-based GDL model employs GIN-style message passing and uses Focal Loss to mitigate class imbalance during training [58].

(8) $$\begin{array}{c} H^{\left(\ell +1\right)}=\sigma \;({\tilde{D}}^{-\frac{1}{2}}\tilde{A}{\tilde{D}}^{-\frac{1}{2}}\\ H^{\left(\ell \right)}W^{\left(\ell \right)}). \end{array}$$

(9) $$\begin{array}{cc} e_{ij} & =\mathrm{LeakyReLU}\;\left(a^{⊤}\left[Wh_{i}\;∥\;Wh_{j}\right]\right),\\ e_{ik} & =\mathrm{LeakyReLU}\;\left(a^{⊤}\left[Wh_{i}\;∥\;Wh_{k}\right]\right),\\ \alpha_{ij} & =\frac{\exp(e_{ij})}{\sum_{k\in N(i)}\exp\left(e_{ik}\right)}. \end{array}$$

(10) $$\begin{array}{cc} m_{v}^{\left(\ell +1\right)} & =\sum_{u\in N(v)}\phi^{\left(\ell \right)}\;\left(h_{u}^{\left(\ell \right)},h_{v}^{\left(\ell \right)},e_{uv}\right),\\ h_{v}^{\left(\ell +1\right)} & =\psi^{\left(\ell \right)}\;\left(h_{v}^{\left(\ell \right)},m_{v}^{\left(\ell +1\right)}\right). \end{array}$$

The Equations (8)–(10) summarize how neighborhood information is condensed on the graph and how node representations are transmitted between layers.

### 3.5. Evaluation Metrics and Statistical Analysis

To comprehensively evaluate the performance of all models in HIV bioactivity prediction, multiple complementary evaluation metrics were employed. These metrics capture different aspects of predictive performance and provide a more robust assessment than relying on a single criterion. The primary evaluation metrics include Accuracy, Precision, Recall, and F1-score, which are defined as follows:

(11) $$\begin{array}{cc} \mathrm{Precision} & =\frac{TP}{TP+FP},\\ \mathrm{Recall} & =\frac{TP}{TP+FN},\\ F_{1} & =\frac{2\cdot \mathrm{Precision}\cdot \mathrm{Recall}}{\mathrm{Precision}+\mathrm{Recall}},\\ \mathrm{Accuracy} & =\frac{TP+TN}{TP+TN+FP+FN}. \end{array}$$

In addition to threshold-dependent metrics, the Area Under the Receiver Operating Characteristic Curve (ROC–AUC) was used as the primary performance indicator. ROC–AUC evaluates the model’s ability to distinguish between active and inactive compounds across all classification thresholds and provides a threshold-independent measure of ranking quality. To ensure robustness and generalizability, all models were evaluated using a 5-fold cross-validation strategy. Performance metrics were reported as mean ± standard deviation across folds, allowing the assessment of both central tendency and variability. Beyond descriptive performance comparison, statistical hypothesis testing was conducted to determine whether observed differences between models are statistically significant. First, the Friedman test was applied as a non-parametric omnibus test to detect overall differences among multiple models. This test evaluates whether at least one model performs significantly differently from the others based on their fold-wise ROC–AUC scores. Following a significant Friedman test result, pairwise comparisons were performed using the paired *t*-test. To control for the family-wise error rate arising from multiple comparisons, Holm correction was applied to adjust *p*-values. In addition to statistical significance, effect size analysis was conducted using Cohen’s *d* to quantify the magnitude of differences between models. This provides further insight into whether observed improvements are not only statistically significant but also practically meaningful. This multi-level evaluation framework, combining performance metrics, statistical testing, and effect size analysis, provides a comprehensive and complementary assessment of model performance. It also strengthens the validity of conclusions drawn in the experimental results section. Also, to quantify sampling uncertainty, ROC–AUC, PR–AUC, and MCC were additionally summarized across 30 fingerprint-matched repetitions using mean ± standard deviation and bootstrap 95% confidence intervals.

## 4. Conclusions

This study presented a comprehensive comparative evaluation of classical machine learning, deep learning, and graph-based models for HIV molecular bioactivity prediction under a unified experimental framework. Unlike many existing studies that focus solely on predictive performance, this study integrates statistical validation with complementary predicted ADME and drug-likeness characterization into the evaluation pipeline.

The experimental results demonstrated that geometric deep learning approaches, particularly the GDL model, achieved the highest discriminative performance (ROC–AUC = 0.956), followed by GRU and RF models, which also exhibited strong and stable results. Statistical analysis indicated overall differences in model performance according to the Friedman test. However, Holm-corrected pairwise comparisons did not demonstrate statistically significant differences between the highest-performing models. These findings should therefore be interpreted as evidence of overall performance differences rather than conclusive statistical superiority. However, it was also observed that high predictive performance does not necessarily translate into pharmacologically viable candidate molecules. The complementary predicted ADME and drug-likeness characterization showed that while some high-performing models (e.g., GDL and GRU) tended to prioritize compounds with more favorable predicted physicochemical, ADME, and drug-likeness characteristics, other models assigned high prediction probabilities to compounds exhibiting multiple drug-likeness constraint violations or less favorable predicted ADME profiles. This finding emphasizes the importance of integrating pharmacokinetic considerations into model evaluation rather than relying solely on classification metrics. The results indicate that the most informative models are not necessarily those with the highest predictive scores, but those that combine strong predictive performance with favorable predicted physicochemical, ADME, and drug-likeness characteristics. Also, molecular docking studies were conducted to evaluate the predicted interaction profile of the GDL1 compound selected by the GDL model with HIV-1 integrase, HIV-1 protease, and HIV-1 reverse transcriptase (RT). The predicted docking scores suggested favorable binding to all three targets, with the most favorable predicted binding score observed for HIV-1 reverse transcriptase. Because docking analysis was performed only for GDL1, these findings should be interpreted as a complementary computational assessment and not as evidence of a correlation between predicted ADME properties and docking performance. The proposed multi-level evaluation framework provides a comprehensive strategy for jointly assessing predictive performance and the predicted molecular characteristics of the highest-ranked compounds from the independent test set. For future work, the integration of larger and more diverse datasets, validation using independent external benchmark datasets, and experimental (in vitro/in vivo) evaluation of the highest-ranked compounds identified by the predictive models would further strengthen the reliability, generalizability, and practical applicability of the proposed framework.

## Figures and Tables

**Table 1:** Summary of related works on HIV molecular bioactivity prediction.

Ref.	Purpose	Method	Dataset /Target	Analysis and Findings
[26]	HIV/HCV multitarget inhibitor prediction	Multiple QSAR (Naïve Bayes + SVM; MACCS & ECFP6 fingerprints)	ChEMBL; 11 HIV-1 and 4 HCV targets	Developed 60 QSAR models (AUC: 0.83–1.00); correctly identified 7 of 9 confirmed HIV/HCV co-inhibitors using complementary NB and SVM models.
[20]	Anti-HIV compound identification via CCR5 inhibition	QSAR-based ML (XGBoost, Histogram-based Gradient Boosting); SHAP; molecular docking; MD simulation	ZINC-22 (37 billion compounds); ChEMBL CCR5 bioactivity data	Trained 40 ML algorithms to screen 37 billion compounds, identifying 124 potential CCR5 inhibitors. XGBoost, HistGBM, LightGBM, and Extra Trees achieved the best performance.
[11]	HIV-1 inhibitor classification from ChEMBL	Hybrid ML/DL pipeline including RFC, KNN, MLP, CNN, and SVC	ChEMBL; 7552 HIV-1 inhibitors; COCONUT (4511 natural compounds)	RFC achieved the highest accuracy (0.9526–0.9932), while 4511 compounds were consistently predicted as active by all three models. The multi-fingerprint strategy improved discriminative performance.
[27]	HIV drug resistance classification	Transfer learning (domain adaptation); mixed HIV dataset balancing	Mixed HIV patient clinical dataset; adverse drug reaction records	Proposed a transfer learning framework for HIV treatment-failure classification, addressing class imbalance and improving early detection.
[28]	HIV-inhibiting molecule generation for virtual screening	Classifier Guidance Diffusion model (Diff4VS); ligand-based virtual screening; DrugIndex metric	MoleculeNet HIV dataset (ligand-based; ∼41,000 molecules)	Introduced the first classifier-guided diffusion model for HIV molecule generation, producing more candidate molecules than baseline methods and proposing the DrugIndex metric.
[29]	HIV activity classification from SMILES	Graph Attention Network (GAT); SMILES molecular representation	MoleculeNet HIV dataset (41,127 molecules)	Applied GAT to molecular graphs for HIV classification, capturing structural topology more effectively than fingerprint-based methods through end-to-end learning.
[12]	HIV drug interaction feature extraction	Mol2Vec algorithm; NLP-inspired molecular embedding; ML classifiers	HIV drug interaction dataset; SMILES-based molecular representations	Employed Mol2Vec embeddings to represent molecular substructures, outperforming sparse fingerprints for HIV drug interaction prediction.
[30]	De novo design of mitotic kinesin Eg5 inhibitors (antitumor/breast cancer)	Message Passing Neural Network (MPNN); LSTM-based transfer learning; bioactivity prediction	ChEMBL Eg5 inhibitor data	Combined MPNN with LSTM transfer learning for bioactivity prediction, improving generalization and enabling de novo Eg5 inhibitor design.
[31]	Molecular property prediction, including HIV classification	Attentive FP (Graph Attention Mechanism); AttentiveFP vs. SVM, RF, MPNN benchmarks	MoleculeNet benchmarks: HIV, BACE, BBBP, Tox21, ToxCast, SIDER, ClinTox	AttentiveFP outperformed SVM, RF, and MPNN on MoleculeNet tasks, achieving state-of-the-art ROC–AUC for HIV classification.
[7]	Neural Message Passing for Quantum Chemistry	Message Passing Neural Network (MPNN); graph-based molecular representation learning	QM9 quantum chemistry dataset	Proposed the unified MPNN framework for molecular property prediction and achieved state-of-the-art results on the QM9 benchmark using iterative message passing on molecular graphs.
[8]	Semi-Supervised Classification with Graph Convolutional Networks	Graph Convolutional Network (GCN); spectral graph convolution; semi-supervised node classification	Cora, Citeseer, PubMed citation networks; NELL knowledge graph	Proposed a simplified first-order Graph Convolutional Network (GCN) for semi-supervised node classification, demonstrating efficient propagation of node features and graph structure while achieving state-of-the-art performance on citation network benchmarks.
[32]	Benchmark framework for molecular ML, including HIV	Comparison of GCN, MPNN, Weave, RF, SVM, and logistic regression across datasets	MoleculeNet suite: HIV, BBBP, Tox21, SIDER, MUV, PCBA, ESOL, FreeSolv, Lipophilicity	Introduced MoleculeNet as a standardized benchmark; on the imbalanced HIV task (scaffold split), Kernel SVM (ROC–AUC ≈ 0.792) outperformed graph convolution (ROC–AUC ≈ 0.763), while graph models were stronger on several other datasets.
[5]	Review of AI applications in HIV research	Systematic review: QSAR, GNN, DL, reinforcement learning, generative models	Literature review across HIV inhibitor, resistance, and drug design studies (2020–2025)	Reviewed recent AI advances in HIV drug discovery, identifying GNNs and transformer-based models as state-of-the-art approaches.
[33]	Molecular descriptor selection for drug bioactivity	Random Forest (RF) with automatic descriptor selection; comparison with SVM and DNN	Ligand datasets for kinases, nuclear hormone receptors, and other enzymes	Demonstrated that RF-based descriptor selection improved predictive accuracy while reducing feature dimensionality.

**Table 2:** Performance analysis (mean ± std) comparison of all models for HIV bioactivity prediction.

Model	Accuracy	Precision	Recall	F1 Score	ROC–AUC
GCN	0.719±0.014	0.710±0.056	0.786±0.110	0.739±0.023	0.813±0.022
GAT	0.679±0.019	0.689±0.033	0.684±0.053	0.685±0.021	0.769±0.024
MPNN	0.663±0.032	0.660±0.033	0.706±0.051	0.681±0.032	0.729±0.042
RNN	0.738±0.220	0.416±0.161	0.491±0.178	0.403±0.086	0.718±0.077
**GRU **	**0.892 ± 0.019**	**0.909 ± 0.056**	**0.821 ± 0.028**	**0.862 ± 0.021**	**0.930 ± 0.017**
**RF**	**0.865 ± 0.035**	**0.944 ± 0.023**	**0.782 ± 0.054**	**0.855 ± 0.041**	**0.927 ± 0.023**
SVM	0.860±0.020	0.886±0.019	0.833±0.023	0.859±0.021	0.915±0.018
MLP	0.840±0.017	0.880±0.017	0.797±0.040	0.835±0.020	0.900±0.022
Mol2Vec+SVM	0.841±0.017	0.901±0.020	0.773±0.030	0.832±0.020	0.907±0.014
CNN	0.506±0.013	0.515±0.013	0.576±0.048	0.543±0.022	0.512±0.017
BRNN	0.505±0.026	0.510±0.023	0.701±0.187	0.584±0.066	0.491±0.026
**GDL**	**0.871 ± 0.026**	**0.997 ± 0.008**	**0.748 ± 0.051**	**0.854 ± 0.032**	**0.956 ± 0.015**

**Table 3:** Selection information for the two highest-ranked compounds prioritized by each predictive model for comparative ADME analysis.

Model	Rank	Selected CV Fold ^†^	True Label	Predicted Class	Predicted Probability	Lipinski Rule
GDL	1	3	1	1	0.934113	Pass
GDL	2	3	1	1	0.922188	Pass
GRU	1	3	1	1	0.999580	Pass
GRU	2	3	1	1	0.999570	Pass
RF	1	5	1	1	1.000000	Pass
RF	2	5	1	1	1.000000	Pass
GAT	1	4	1	1	0.966774	Pass
GAT	2	4	1	1	0.954624	Pass
GCN	1	5	1	1	0.945309	Pass
GCN	2	5	1	1	0.950407	Pass
MPNN	1	4	0	1	0.795909	Pass
MPNN	2	4	1	1	0.803431	Pass
MLP	1	2	1	1	0.991311	Pass
MLP	2	5	1	1	0.988675	Pass
Mol2Vec+SVM	1	1	1	1	0.999993	Fail
Mol2Vec+SVM	2	1	1	1	0.999995	Fail
SVM	1	2	1	1	0.991311	Pass
SVM	2	5	1	1	0.988675	Pass
BRNN	1	3	0	1	0.559672	Pass
BRNN	2	3	0	1	0.560834	Pass
RNN	1	2	1	1	0.626272	Pass
RNN	2	2	1	1	0.668684	Pass
CNN	1	1	0	1	0.518682	Pass
CNN	2	1	0	1	0.519083	Pass

^†^ Cross-validation fold selected according to the highest validation ROC–AUC within the development set. The independent hold-out test set was not used for fold selection.

**Table 4:** Overlap analysis of the highest-ranked compounds selected by different predictive models.

Canonical SMILES	Selected by Models	No. of Models
O=[N+]([O-])c1ccccc1S(=O)(=O)c1cc(Cl)cc2c1NCCC2	SVM, MLP, GCN	3
CCNc1ccccc1S(=O)(=O)c1ccccc1[N+](=O)[O-]	SVM, MLP	2

**Table 5:** Maximum fingerprint similarity between the selected compounds and the corresponding training compounds together with the label of the nearest training analogue.

Model	Candidate	Maximum Tanimoto Similarity	Nearest Training Analogue Label
GDL	1	0.556	Active (1)
GDL	2	0.778	Active (1)
GRU	1	1.000	Active (1)
GRU	2	1.000	Active (1)
RF	1	0.754	Active (1)
RF	2	1.000	Active (1)
GAT	1	0.774	Active (1)
GAT	2	1.000	Active (1)
GCN	1	0.744	Active (1)
GCN	2	1.000	Active (1)
MPNN	1	1.000	Inactive (0)
MPNN	2	1.000	Active (1)
MLP	1	0.760	Active (1)
MLP	2	0.647	Active (1)
Mol2Vec+SVM	1	1.000	Active (1)
Mol2Vec+SVM	2	1.000	Active (1)
SVM	1	0.760	Active (1)
SVM	2	0.647	Active (1)
BRNN	1	1.000	Inactive (0)
BRNN	2	0.514	Inactive (0)
RNN	1	1.000	Active (1)
RNN	2	0.698	Active (1)
CNN	1	1.000	Inactive (0)
CNN	2	1.000	Inactive (0)

**Table 6:** Sensitivity analysis of Random Forest performance using chemically matched inactive subsets.

Metric	Original HIV Subset	Fingerprint-Matched Subsets	Bootstrap 95% CI
		(Mean ± SD)	
ROC–AUC	0.7562	0.7685±0.0060	0.7664–0.7706
PR–AUC	0.8124	0.8236±0.0038	0.8223–0.8249
MCC	0.3234	0.4092±0.0157	0.4036–0.4146

**Table 7:** Chemical similarity between the archived HIV inactive subset and the fingerprint-matched alternative subsets.

Property	Value
Number of repetitions	30
Inactive compounds per repetition	1412
Mean Tanimoto similarity	0.4856
Median Tanimoto similarity	0.4722
Tanimoto IQR	0.3421–0.6098
Mean same-scaffold rate	0.6314

**Table 8:** Statistical results of ROC–AUC-based pairwise comparisons between the GDL model and all other models.

Comparison	Base Mean	Other Mean	Mean Diff	*p*- Value	Holm *p*	Cohen’s d	Significance
GDL vs. BRNN	0.9560	0.4909	+0.4652	<0.001	<0.001	35.34	Significant
GDL vs. CNN	0.9560	0.5120	+0.4441	<0.001	<0.001	17.43	Significant
GDL vs. RNN	0.9560	0.7179	+0.2381	0.0018	0.0108	2.74	Significant
GDL vs. MPNN	0.9560	0.7291	+0.2270	<0.001	<0.001	5.51	Significant
GDL vs. GAT	0.9560	0.7688	+0.1873	<0.001	<0.001	13.53	Significant
GDL vs. GCN	0.9560	0.8126	+0.1434	<0.001	<0.001	7.72	Significant
GDL vs. MLP	0.9560	0.8996	+0.0564	0.0042	0.0170	2.17	Significant
GDL vs. Mol2Vec+SVM	0.9560	0.9073	+0.0488	0.0034	0.0170	2.30	Significant
GDL vs. SVM	0.9560	0.9153	+0.0408	0.0209	0.0533	1.32	Not significant
GDL vs. RF	0.9560	0.9274	+0.0287	0.0616	0.0616	0.87	Not significant
GDL vs. GRU	0.9560	0.9296	+0.0265	0.0178	0.0533	1.40	Not significant

**Table 9:** Druglikeness, water solubility and pharmacokinetic properties of all compounds.

Model	Druglikeness					Water Solubility		Pharmacokinetics	
Comp No	Lipinski	Ghose	Veber	Egan	Muegge	LogS	Class	GI abs.	F
SVM1	+	+	+	+	+	−4.45	Moderately	High	0.55
SVM2	+	+	+	+	+	−4.01	Moderately	High	0.55
RF1	+	−	+	+	+	−5.77	Moderately	High	0.55
RF2	+	+	+	+	+	−2.06	Soluble	High	0.55
MLP1	+	+	+	+	+	−3.38	Soluble	High	0.55
MLP2	+	−	+	−	+	−5.78	Moderately	Low	0.55
BRNN1	+	+	+	+	+	−3.82	Soluble	High	0.55
BRNN2	+	+	+	+	+	−2.84	Soluble	High	0.55
CNN1	+	+	+	+	+	−3.57	Soluble	High	0.55
CNN2	+	+	+	+	+	−3.22	Soluble	High	0.55
Mol2Vec-1	+	−	−	−	−	−6.61	Poorly	Low	0.11
Mol2Vec-2	−	−	−	−	−	−5.26	Moderately	Low	0.17
GDL1	+	+	+	+	+	−4.00	Soluble	High	0.56
GDL2	+	+	+	+	+	−3.41	Soluble	High	0.55
GRU1	+	+	+	+	+	−4.99	Moderately	High	0.55
GRU2	+	−	+	−	+	−5.78	Moderately	Low	0.55
RNN1	+	+	+	+	+	−2.95	Soluble	High	0.55
RNN2	+	+	+	+	+	−2.13	Soluble	High	0.55
GAT1	+	+	+	+	+	−3.68	Soluble	High	0.55
GAT2	+	+	+	+	+	−3.58	Soluble	High	0.55
GCN1	+	+	+	+	+	−3.77	Soluble	High	0.55
GCN2	+	+	+	+	+	−4.48	Moderately	High	0.55

**Table 10:** The physicochemical and lipophilicity properties of all compounds.

Model	Physicochemical Properties							Lipophilicity
Comp. No	MW	Fsp3	RB	HBA	HBD	MR	TPSA	cLogP
SVM1	352.79	0.20	3	4	1	92.11	100.37	2.79
SVM2	306.34	0.14	5	4	1	81.31	100.37	2.24
RF1	485.48	0.36	10	3	1	128.02	64.09	3.44
RF2	311.33	0.71	3	6	2	81.49	96.79	0.67
MLP1	332.33	0.07	4	6	2	93.21	128.25	1.71
MLP2	457.28	0.14	4	7	1	97.34	123.54	4.05
BRNN1	236.27	0.07	2	3	1	69.80	54.56	2.63
BRNN2	214.31	0.50	1	2	0	69.39	27.03	2.38
CNN1	250.75	0.25	1	1	0	71.62	45.53	3.19
CNN2	237.36	0.54	3	2	2	70.21	71.06	2.84
Mol2Vec-1	563.34	0.09	5	9	4	128.52	174.65	3.39
Mol2Vec-2	581.66	0.73	6	10	2	149.91	177.44	2.80
GDL1	355.84	0.38	6	4	1	91.97	89.93	3.24
GDL2	386.53	0.64	7	4	1	124.19	55.89	2.28
GRU1	452.53	0.26	7	6	2	128.41	123.76	3.42
GRU2	457.28	0.14	4	7	1	97.34	123.54	4.16
RNN1	235.31	0.45	0	2	2	65.66	89.88	2.15
RNN2	281.29	0.58	3	6	1	70.20	76.30	0.65
GAT1	277.30	0.08	3	4	0	72.17	88.34	2.29
GAT2	261.30	0.08	3	3	0	71.48	82.10	2.38
GCN1	292.31	0.08	4	4	1	76.51	100.37	1.93
GCN2	352.79	0.20	3	4	1	92.11	100.37	2.79
MPNN1	230.20	0.08	0	2	1	65.25	47.11	2.14
MPNN2	459.45	0.00	4	10	3	111.61	183.34	2.75

Comp: Compounds, Fsp3: Fraction Csp3, RB: Number of rotatable bonds, MR: Molar refractivity.

**Table 11:** Estimated inhibition constants and docking scores of the GDL1 compound against three major HIV-1 therapeutic targets.

Target (PDB ID)	Estimated $K_{i}$	AutoDock4	AutoDock Vina
		Dock Score	Dock Score
HIV-1 Protease (1AJV)	665.18 nM	−8.43	−8.0
HIV-1 Integrase (1QS4)	29.40 μM	−6.18	−5.6
HIV-1 Reverse Transcriptase (2ZD1)	163.49 nM	−9.26	−7.9

Dock score represents the predicted binding free energy (kcal/mol). $K_{i}$ denotes the estimated inhibition constant. nM: nanomolar; μM: micromolar.

**Table 12:** Compound selection protocol for comparative ADME analysis.

Item	Description
Source partition	Independent hold-out test partition
Performance estimation	Stratified five-fold cross-validation
Fold selection criterion	Highest test ROC–AUC
Prediction source	Trained model of the selected fold
Compound ranking criterion	Predicted probability ($y_{prob}$)
Model-level metrics	None (ranking based exclusively on $y_{prob}$)
Aggregation across folds	None
Selected compounds	Two highest-ranked compounds per model

**Table 13:** Hyperparameter configuration of all models.

Model	Loss	Optimizer	LR	WD	Dropout	Epochs	Batch	Representation/Architecture
SVM (Morgan FP)	hinge (SVC)	—	—	—	—	—	—	Morgan *r* = 2, 2048 bits; StandardScaler; SVC(rbf); *C*, γ default
RandomForest (Morgan FP)	Gini	—	—	—	—	—	—	Morgan *r* = 2, 2048 bits; n_estimators = 100, n_jobs = −1
MLP (Morgan FP)	log-loss	Adam	—	$10^{-4}$	—	200	—	Morgan *r* = 2, 2048 bits; hidden_layer_sizes = (100,); max_iter = 200
Mol2Vec+SVM	SVC (hinge-based)	—	—	—	—	—	—	Mol2Vec embeddings (300-D); StandardScaler; SVC(RBF) C = 1.0, γ = scale)
BRNN (Keras)	binary_crossentropy	Adam	$10^{-3}$	L2 $10^{-4}$	0.2 / 0.0	200	128	Bidirectional SimpleRNN(64)+Dense; ReduceLROnPlateau, min_lr = $10^{-5}$
RNN (Keras)	binary_crossentropy	Adam	$5\cdot 10^{-4}$	—	0.40	40	64	Embedding(64)+SimpleRNN(64); MAX_LEN = 32
CNN (Keras)	binary_crossentropy	Adam	$10^{-3}$	—	—	200	128	Two Conv1D(128, *k* = 3)+Dense; class_weight balanced
GRU (Keras)	binary_crossentropy	Adam	$5\cdot 10^{-4}$	—	0.40 / 0.15 rec.	40	32	Embedding(32)+GRU(32); MAX_LEN = 64
GAT (PyTorch Geo.)	BCELoss	Adam	$3\cdot 10^{-4}$	$10^{-4}$	0.3 / 0.4	120	32	GATConv 128→64, heads = 4; ReduceLROnPlateau
GCN (PyTorch Geo.)	wBCE / BCELoss	Adam	$3\cdot 10^{-4}$	$10^{-4}$	0.35	50	64	3×GCNConv+MLP head
MPNN / GIN (PyTorch Geo.)	BCELoss	Adam	$5\cdot 10^{-4}$	$10^{-4}$	0.3	80	32	Two GINConv(64)+Linear; early stop patience = 10
GDL (PyTorch)	sigmoid focal	AdamW	$10^{-4}$	$10^{-3}$	0.2	60	128	GIN-style; HIDDEN_DIM = 128, N_LAYERS = 4; focal α = 0.25, γ = 2.0

## Data Availability

The HIV dataset used in this study is publicly available as part of the MoleculeNet benchmark. The processed dataset, source code, model implementations, software environment information, and outputs generated during this study are publicly available at: https://github.com/aycabostancioglu/HIV-ADME-Framework (accessed on 9 June 2026).

## Abbreviations

The following abbreviations are used in this manuscript:

ADME	Absorption, Distribution, Metabolism, and Excretion
AI	Artificial Intelligence
AIDS	Acquired Immunodeficiency Syndrome
AUC	Area Under the (ROC) Curve
BRNN	Bidirectional Recurrent Neural Network
CADD	Computer-Aided Drug Design
cLogP	Consensus Log P (average of five predictions)
CNN	Convolutional Neural Network
DL	Deep Learning
DNN	Deep Neural Network
ECFP	Extended-Connectivity Fingerprint
GAT	Graph Attention Network
GBDT	Gradient Boosting Decision Tree
GCN	Graph Convolutional Network
GDL	Geometric Deep Learning
GIN	Graph Isomorphism Network
GNN	Graph Neural Network
GRU	Gated Recurrent Unit
HBA	Hydrogen Bond Acceptors
HBD	Hydrogen Bond Donors
HIV	Human Immunodeficiency Virus
LogP	Octanol–Water Partition Coefficient
LSTM	Long Short-Term Memory
MACCS	Molecular ACCess System structural keys
ML	Machine Learning
MLP	Multi-Layer Perceptron
MPNN	Message Passing Neural Network
MW	Molecular Weight
MoleculeNet	Standard molecular machine learning benchmark suite (MoleculeNet)
PRC	Precision-Recall Curve
QED	Quantitative Estimate of Drug-likeness
RBF	Radial Basis Function
RF	Random Forest
RNN	Recurrent Neural Network
ROC	Receiver Operating Characteristic
SMILES	Simplified Molecular-Input Line-Entry System
SVM	Support Vector Machine
TPSA	Topological Polar Surface Area
